# Development and validation of a Database Forensic Metamodel (DBFM)

**DOI:** 10.1371/journal.pone.0170793

**Published:** 2017-02-01

**Authors:** Arafat Al-dhaqm, Shukor Razak, Siti Hajar Othman, Asri Ngadi, Mohammed Nazir Ahmed, Abdulalem Ali Mohammed

**Affiliations:** 1 Department of Computer Science, Faculty of Computing, Universiti Teknologi Malaysia, Skudai, Johor, Malaysia; 2 Department of Computer Science, Aden Community College, Aden, Yemen; University of Texas at San Antonio, UNITED STATES

## Abstract

Database Forensics (DBF) is a widespread area of knowledge. It has many complex features and is well known amongst database investigators and practitioners. Several models and frameworks have been created specifically to allow knowledge-sharing and effective DBF activities. However, these are often narrow in focus and address specified database incident types. We have analysed 60 such models in an attempt to uncover how numerous DBF activities are really public even when the actions vary. We then generate a unified abstract view of DBF in the form of a metamodel. We identified, extracted, and proposed a common concept and reconciled concept definitions to propose a metamodel. We have applied a metamodelling process to guarantee that this metamodel is comprehensive and consistent.

## Introduction

Database Forensics (DBF) is a field of digital forensic investigation that addresses database contents and their metadata [1]. It is considered a significant field by which to identify, detect, acquire, analyse, and reconstruct database incidents and reveal intruders’ activities. DBF has suffered from several issues, which has resulted in it becoming a heterogeneous, confusing and unstructured domain. Examples of these issues include a variety of database system infrastructures; the multidimensional nature of database systems; and domain knowledge effectively being scattered in all directions [2,3]. A variety of database system infrastructures with multidimensional natures has enabled the DBF domain to address specific incidents. Therefore, each database management system (DBMS) has a specific forensic investigation model/approach. Consequently, the issues of different concepts and terminologies in terms of the forensic investigation process and the scattering of domain knowledge in all directions have produced other challenges for DBF investigators and practitioners. This knowledge (such as models, processes, techniques, tools, frameworks, methods, activities, approaches, and algorithms) is neither organized nor structured. Furthermore, it is universally dispersed, such as in the Internet, books, journals, conferences, online databases, book chapters, dissertations, reports, and organizations. Consequently, there is a lack of generic/standardized models by which to unify concepts and terminologies that may be used to reduce confusion and assist in organizing and structuring domain knowledge. This study discusses the DBF domain from several perspectives to highlight, extract, compare, merge and derive common concepts of the domain as well as to harmonize and reconcile concepts and definitions, such as i) the Database Dimensions perspective; ii) Database Forensic Technology perspective; and iii) Database Forensic Investigation process perspective.

This study applies a metamodelling approach to unify existing attempts to represent DBF knowledge in a reusable form and provide a unified viewpoint of access. Metamodelling has been achieved by way of the Object Management Group (OMG) [4]. We explain our unification method by offering common concepts and reconciled definitions that generalize most of the concepts used in existing DBF practices as described in existing models. This paper is structured as follows: In Section 2, we present some works related to this research. Section 3 provides the actual development process of our DBF metamodel based on a metamodelling approach. Section 4 presents the validation of the proposed DBF Metamodel. Section 5 presents the limitations of the DBF Metamodel. Finally, we conclude this paper in Section 6 with recommendations for possible future work related to this paper.

## 2.0 Backgrounds and related work

Several types of modelling languages have been offered for various disciplines, including business process modelling [5]; systems engineering; and software engineering [6,7]. These languages are naturally used to identify systems that can provide a better understanding for stockholders. This study targets the emergence of a modelling language to define the domain of DBF. In addition, it draws on research from the field of metamodelling [8,9] to develop a process by which to create such a language. Generally, a metamodelling process aims to generate a group of *classes* to represent domain entities and define domain concepts, actions or states [10]. This concept group is called the *metamodel*. The language that we look for is supported by the *metamodel*. It has the ability to generalize the domain by gathering all field/domain concepts and dividing the field/domain issues into sub-domain issues. A more difficult mission in the development of a domain description is to determine how the end user will form his/her own model with the concepts and notation from a domain *language*. In the field of software engineering, a metamodel targets the generation of portable software actions and components. Further, it is interoperable and reusable. A metamodel also contains the description of the particular modelling environment for a firm domain and describes the syntax and semantics of the domain. It can be viewed from the following three different perspectives: i) as a set of structuring blocks and rules used to construct and control new models; ii) as the model of a domain of interest; and iii) as an instance of another model. In our situation, a metamodel is an essential structuring block that creates statements about the possible structure of DBF models [11].

Several metamodelling frameworks have been defined by many information systems researchers, e.g., [17–24]. In this study, we follow a metamodelling framework based on the Meta Object Facility (MOF) [12] presented by OMG. Our DBF metamodel (DBFM) will comprise a set of concepts of the DBF Language and their relationships matching with the Metamodel layer of MOF. Through the use of DBFM, it will be easier to manage multiple requirement perspectives as supported in [13,14]. DBFM will identify the relationships existing between DBF models and indirectly define possible relationships between the various perspectives described by the models. This study will develop an iterative metamodelling process to ensure that it becomes domain-independent. In other words, the resultant process will not require an in-depth knowledge of DBF to enable the production of the metamodel. We will apply it to generate a complete and comprehensive DBF metamodel, which will be the final product of an iterative process. Its evolution will be interleaved with the validation process of the metamodel. Any improvements resulting from the validation process (e.g., concept improvement/deletion/addition) will directly feed into the iterative process. In [15], they identified a number of techniques that use external sources to validate the concepts in the metamodel (e.g., other existing models or DBF descriptions). Specifically, we illustrated the ‘*Comparison against other models’* [16,17] using one external source to validate a part of the preliminary version of the metamodel. For the purpose of the validation in this study, we refine 18 DBF models in detail by using our metamodel and applying multiple validation techniques.

The quality of the metamodel is measured based on how well it can achieve the purpose of its development [18,19], specifically by addressing the needs of domain practitioners; increasing the transparency of the knowledge encoded within the domain applications; and being amenable to validation by experts in the domain area. Our *end users (domain practitioners)* include a database administrator, a DBF investigator and/or response managers for various public and private database organizations seeking to create a DBF model to manage expected database incidents. Database Forensics (DBF) includes all features of preparation and response to all processes of database forensic investigation, including *identification*, *collection and preservation*, *analysis*, *and presentation* activities.

This study targets a reduction of interoperability challenges and facilitates the process of knowledge-sharing similar to [20], but in DBF. It is common that no two database incidents are precisely the same and that every database incident needs its own managing process. However, the ways in which database incidents affect database integrity and business continuity are similar, and responses are often transferrable between database incidents. For example, a *collection of volatile data* is a DBF action that is applicable in various DBF situations [1]. We use a variety of models and frameworks that have been developed by many domain experts, investigators, or companies on the subject of DBF studies. Existing DBF models can be categorized as *requirements models* because of abstract representations of an existing or a desired model in the real world (e.g., detection tampering [21], preparation and verification [22], technology tools [23], decision-making [24], response and identification [25], collection and analysis [26], and reconstruction evidence [27]). The meaning and definition of specific concept terminologies and their relationships may differ from one observer to another [28]. Domain concepts can have multiple descriptions. Some concepts are observed to represent similar DBF activities that are expressed differently. For example, in a System and Method for Investigating a Data Operation Performed on a Database [23], the terminology ‘*Reconstructing Database’* is used to reconstruct the database events from logs and memory caches. The same activity, however, is represented using ‘*Rebuilding’* in the Database Application Schema Forensics Model [29]. A specific domain modelling language expressed as a metamodel can offer an alternative and better approach towards resolving this type of problem. Our approach unifies the various terminologies used. The DBF Metamodel (DBFM) developed in this study describes all the DBF model concepts and the way they are arranged/organized, linked and constrained. It also provides a flexible structure by which to facilitate the storage and retrieval of DBF knowledge. This work is extended to our works published between 2014 and 2016 [30–32].

Therefore, DBF domain has been discussed from three perspectives: i) Database Forensic Dimensions -based (e.g., destroyed, compromised, and changed); ii) Database Forensic Technology-based (e.g., tools, algorithms, and methods); and iii) Database Forensic Investigation Process-based (e.g., Identification, Artefact collection, Artefact analysis, Documentation and Presentation). Compromised database define as a database where some of the metadata or some software of the database management system (DBMS) have been modified by an attacker even though the database is still operational. Modified database: In contrast to compromised databases, the category of damaged or destroyed databases refers to databases where the data contained or other data files may have been modified, deleted or copied from their original locations into other places. These databases may or may no longer be operational depending on the extent of the damage done. Most of the research in database forensics falls into this category. *Damaged database*: We refer to a modified database as a database which has not been compromised or damaged but has undergone changes due to normal business processes since the event of interest occurred. This category of databases is often of interest when a database is not directly involved in the crime being investigated but is used to store information that may assist in solving other crimes.

On the other perspective (forensic technology perspective), we were discovering from the literature review that most of the models are focusing on the technical side (specific tools, algorithms, methods). For example [23] have been offered specific forensic technology and forensic mechanisms to reveal Oracle database activities such as Log Miner, Flashback, Recycle Bin, and System Change Number (SCN), Undo log and Redo log. Also, [33] offered a forensic tool called “Log Miner” to analyse Oracle database activities. Additionally, Litchfield offered series of technical models [25,34–39] to deal with several specific incidents, cases and scenarios of Oracle database. Furthermore, several studies such as [1–3,22,24,26,27,29,40–66], discussed Database Forensic from technology perspectives. For example, methods to detect database tampering, detect covert database server, discovering who is criminal, when crime happen, what and where crime happen, protect evidence methods, acquisition methods, analysis methods, forensic analysis algorithms, etc. have been offered to deal with Database Forensic. Consequently, no of the offered models has been covered whole Database Forensic domain. Some models deal with Oracle database forensic, and some deal with MSSQL Server forensic, where others deal with MySQL database forensic, DB2 forensic and SQLite forensic. However, they are sharing in some investigation concepts and terminologies. According to [64] **r**esearch in digital forensics has led to the development of various techniques and process models. However, many of these techniques are not completely transferable to database forensics due to certain characteristics of databases which require them to be adapted for handling database forensics.

Finally, the Database Forensic has been discussed from investigation process perspective. Therefore, Investigation process model for Oracle database was offered by [23] to reveal malicious database activities. It consists of, four investigation process: *Suspend database operation*, *collecting data*, *reconstructing database*, *and restoring database integrity*. Another model was developed by [33] to discover and analyse intruder activities in MSSQL server database. It consists of five investigation process namely *Verification*, *Evidence Collection*, *Timeline Creation*, *Media Analysis*, and *Data Recovery*. Also, two investigation processes were extracted from the model [25], namely *Identification*, *and Collection process* to identify and collect the volatile and non-volatile data. Additionally, four investigation processes have been extracted from the model [22] to identify, verify, collect and analyse MSSQL server database incidents. The extracted investigation processes are *Investigation preparation*, *Incident verification*, *Artefact collection*, *and Artefact analysis*. Also, two investigation processes extracted from the model: *Database Connection Environment and Extraction data* to detect and extract malicious relations amongst tables. Other three investigation process: *Data Acquisition*, *Beginning of Investigation*, *and Financial and Business Data Analysis* were offered by [44] to detect fraud statements. Furthermore, [1] offered four investigation processes to extract and search metadata: *Metadata extraction*, *Integrity*, *Restoration*, *and Searchability*. Also, process investigation model has been introduced by [67] in the enterprise environment. It has clear three steps to be taken during the investigation process starting from *detection server* process after the incident reported, followed by *data collection*, *and investigation on data collected* respectively. Also, a tamper detection model has been offered by [52] to introduce digital evidence against database tamper detection. Thus two investigation processes have been highlighted in this model *Setup evidence collection server and Collect Oracle file*.

The framework proposed by [3] to deal with MySQL server database. Generally, it consists of three main investigation processes: *Identification*, *Artefact collection*, *and Artefact analysis*. Another framework has been offered by [68] to analyze database incidents. It consists of six investigation processes: *Incident reporting*, *Examination preparation*, *Physical & digital examination*, *Documentation & Presentation*, *Post examination*, *Post examination analysis*. Also, seven investigation processes have been suggested by [27] to investigate database incidents namely: *Determine database dimension*, *determining acquisition method*, *a collection of volatile artefacts*, *Collection of non-volatile artefacts*, *preservation and authentication of collected data*, *analysis of collected data*, *reconstruction of the database*.

Recently, several works have been offered in digital forensic. However, they are mostly focusing on cloud forensic and smartphone forensic [69–86]. They mentioned Database Forensic in an indirect way.

## 3.0 Metamodelling database forensics

To construct DBFM, a group of common and repeatedly used DBF concepts is first determined. The concepts and their definitions of DBF are listed in the existing DBF literature. A survey of the DBF field/domain is first conducted by studying the huge amount of existing DBF models, frameworks, methods, approaches and techniques from three perspectives (60 in total). This gives us a broad knowledge of DBF actions, activities, and operations. The relationships are used with related common concepts. The metamodel construction development is iterative with nonstop modification of new concepts. To create the DBFM, we used the 8 *steps Metamodelling Creation Process* adapted from [87,88], which is described below.

### 3.1 Preparing knowledge sources

This step involves gathering together the knowledge sources to be used. We have undertaken a meta-study to learn how to distinguish between them. This also enhances our domain awareness as recommended in [89] as an initial step for any metamodelling process. In total, we collected 60 DBF models from a variety of the following sources: journals; conference papers; organization and investigation agencies’ organization reports; online investigation-related websites; books; etc. Collecting these models was performed as follows: we focused on discovering categories of DBF models and ascertained that there are sufficient DBF models in the literature to enable metamodelling to be a feasible path. We used the following academic collections of journals: Scopus; Web of Science; IEEE; Springer; Engineering Village and Google Scholar. For this purpose, we used the following search keywords: ‘database forensic’; ‘detect covert database’; ‘detect database tampering’; ‘analysis database forensic’; ‘database forensic artefacts’; ‘database forensic investigation’; ‘database forensic process’; and ‘database tampering’. This effort led to the discovery of 60 models, frameworks, activities, approaches, mechanisms, and case studies. With this number, we became more confident that the literature on DBF modelling is sufficiently mature to apply a metamodelling process. We categorized these 60 models according to the following three different primary perspectives: i) Database Forensic Dimensions-based (e.g., destroyed, compromised, and changed); ii) Database Forensic Technology-based (e.g., tools, algorithms, and methods); and iii) Database Forensic Investigation Process-based (e.g., Identification, Artefact collection, Artefact analysis, Documentation and Presentation).

Collected in Step 1, two sets of models have been filtered through for the metamodelling-based synthesis of DBFM. This is carried out as follows. Set I is used to initiating the metamodelling process, and this includes 18 models that cover three perspectives of DBF. Another set, Set II, is used to undertake validation of the DBFM (Step 8 in the process). The sets are formed according to how broadly they cover the three perspectives of DBF. Some models cover all three perspectives, some cover 2 perspectives, while others focus on only one perspective. Some models focus on a specific DBF perspective and do not pay too much attention to the boundaries of the DBF perspectives (e.g., reconstruction events (technology-based)). If a model does not cover any DBF perspective, we exclude it from any further investigation. The models included in each set are shown in (Table A in S1 Appendix I).

For Set I, we require wide coverage across the concepts as our aim is to create a DBFM that can be widely applicable. Using the coverage measure alone, we quickly gain an indication of how widely applicable the sourced model is. The model is said to have a high coverage value if it can cover the entire perspective of DBF (*general model*), whereas a model has a lower coverage value if it only describes a specific DBF perspective, such as forensic techniques (*specific model*). As supported by Kelly *et al*. in their discussion regarding practices for the development of domain-specific modelling, “*Finding the proper generic-specific balance is a key-success factor in domain-specific modelling development…” (*[89] *pp*. *25)*. For example, the ‘SQL Server Forensic Analysis Methodology’ [22] could cover most of the DBF aspect in the model, whereas ‘Reconstruction in Database Forensic’ [64] covers only a small portion of the DBF domain. In the selection of models for Set I, we ensured that selected models can cover three (3) perspectives in DBF (Database Forensic Dimensions, Database Forensic Technology, and Database Forensic Investigation Process). The initial metamodel development requires the combination of all generic concepts existing in the domain. The combination of concepts that come from all DBF perspectives will provide generic concepts for our DBFM.

### 3.2 Recognize and extract general concepts

Extraction of concepts from a textual model must comply with tight conditions to avoid any missing or unrelated concepts that may cause doubt or confusion later. Therefore, the concepts should be extracted from the main body of the textual model; this means excluding the title, abstract, introduction, related works, and conclusion. The main body of the textual model explains the perspective of researchers, developers, or experts as to the main clue of this model. For example, Lee’s model “A Workflow to Support Forensic Database Analysis” [24] is a workflow model for Database Forensics that offers six (6) processes; hence, the authors focus on the related concepts of these processes. Additionally, Fowler’s model, “SQL Server Forensic Analysis Methodology” [22], offered forensic methodologies that covered the Microsoft SQL Server Database and proposed four processes, namely *Investigation preparedness; Verification incident; Artefact collection; and Artefact analysis*.

Therefore, authors recognize the concepts contained under these processes. With regard to the other criteria, recognized concepts must be nouns or (adjective + noun). For example, *Database server*, *Investigation Team*, and *Forensic Technique* are nouns, whereas *Volatile Artefact*, and *Non-volatile artefact* is “adjective+noun”. Additionally, the third criterion that may follow excludes any concept that is not related to the domain. According to [90], the golden rule for selecting concepts is “if it is not relevant to the domain, then do not include it in the case domain model”. The case domain model should not be a comprehensive representation of all entities/concepts involved in a case. Rather, the case domain model should represent all case concepts that are essential to the Database Forensics examination. In large-scale cases, it would be especially counterproductive to model every involved concept; even relatively simple cases could yield an unmanageable case domain model if all concepts are exhausted. Thus, each step in the process of constructing a case domain model must be supported by methods and heuristics suitable to the selection of appropriate concepts. Finally, the last recognized criterion is the exclusion of specific concepts related to particular fields, such as Oracle Flashback Transaction; Oracle LogMiner; Oracle tools; SQLedit; MySQL utilities; and so on.

Therefore, in accordance with the findings of previous studies [91–93], and [88], the authors manually extracted concepts from each model (containing 18 models in total). This is a laborious process whereby every model is used to identify potential concepts that are required in this study. The mechanism that was followed to extract concepts is as follows: “read a main textual model word by word to first understand the purpose of the model and then the meaning of each paragraph”. Therefore, the concepts related to the Database Forensic domain should be highlighted and extracted. According to [90], “It is important, to begin with a very exhaustive list of concepts and gradually eliminate concepts that are irrelevant”.

Wong’s model [23] covered the Oracle database server forensic concepts to reveal malicious activities in a database. However, it is limited in scope to the discovery of the SQL injection attack. A total of 17 concepts have been extracted, namely *Capture evidence*, *Intruder activity*, *Database server*, *Collected data*, *Database contents*, *Reconstructing database*, *Redo log*, *Undo log*, *Audit trail*, *Alert log*, *Hashing*, *Examination*, *Backup*, *Transaction*, *Investigation Team*, *Resources*, and finally, *Damage Database*.

Fowler’s model [94] covered MSSQL Server forensic concepts which were used during the investigation process. A total of 17 concepts were extracted from Flower’s model, and these included *Data acquisition*, *Database server*, *Capture*, *Acquired data*, *Transaction logs*, *Evidence integrity*, *Suspicious transaction*, *Evidence*, *Volatile data*, *Non-volatile data*, *Event*, *Output file*, *Log file*, *Database files*, *Incident response*, *Trusted forensic workstation*, *and Timeline*.

Additionally, some 16 further concepts have been identified from Leitchfield’s model [25], including *Redo log*, *Gathering evidence*, *Hashing*, *Examination*, *Forensic examiner*, *Suspicious activity*, *Volatile evidence*, *Non-volatile evidence*, *Incident*, *Database Server*, *Output file*, *Organization*, *Court*, *Live response*, *Collection server*, and *Decision*. Furthermore, 10 concepts were extracted from Olivier’s model [1]: *Database contents*, *Examination*, *Backup*, *Investigation Team*, *Source*, *Evidence*, *Incident*, *DBMS*, *Court*, and *Capture*. (Table B in S2 Appendix I) displays the extracted concepts. Hence, the output of this step is 246 general concepts.

### 3.3 Nominate and propose common concepts

In this step, we nominate common concepts from among 246 extracted concepts based on similarity in meaning or function. Thus, authors nominate the concepts in clusters. Each cluster has similar concepts in meaning and function. The outcome of this process resulted in 44 clusters, as shown in (Table C in S3 Appendix I).

For example, the following are concepts that are clustered based on similar meaning: “*Reconstructing database”* in model [23]; “*Reconstruction*” in models [22], [3], [27], [95,96]; “*Reconstruction event*” in model [60]; and “*Reconstructing*” concept in model [66]. To provide another example, the “*Capture*” concept has been mentioned in three models [94], [97], [24], together with its synonym “*Seizure*” mentioned in the model [44]. These have all been grouped in a cluster. Additionally, the “*Transaction*” concept mentioned in four models [23], [3], [26], [95], [66] has been grouped into one cluster. Therefore, the concepts that have a similar meaning or function have been grouped together in clusters.

Additionally, to propose a common concept for each cluster, we used the following three features to distinguish amongst cluster concepts: *Frequency*, *Generality*, and *Definition*. Thus, a concept that has high frequency, generic meaning and definition may be proposed as a common concept, as illustrated in (Table C in S3 Appendix I). For example, *“Capture”* has been proposed as a common concept in Cluster 1 due to its having higher frequency and definition, as well as covering the entire DBF domain. Additionally, *“DataAcquisition”* has been proposed as a common concept in Cluster 2, along with *“InvestigationTeam”* in Cluster 13. (Table C in S3 Appendix I) displays the proposed common concepts.

### 3.4 Short-listing of candidate definitions

For every concept, we short-list several definitions to use towards deriving a common definition. When two or more concepts share the same definition, a process to reconcile and fit the definition is required. A greater weight is given to sources with clearer definitions (as opposed to those considered implicit definitions that can be subject to interpretation). However, the concepts that have multiple definitions comply with the reconciliation process [87,88], [98]. For example, from the Ideal Log Setting for Database Forensics Reconstruction [96], we short-list only the following concept definitions:

- **Reconstruction**: ‘its application in database forensics also involves the reconstruction of data that might have existed on a database at an earlier time prior to some modifications or deletion’.
- **Transaction logs**: ‘transaction logs are useful for recovery from failed transactions and retrieval of a consistent version of the database in the event that the system crashes’.
- **Log file**: ‘the log files keep a record of the events that occur in the database’.
- **Timeline**: ‘creating a timeline of events can assist an investigator to gain an insight into the events that occurred as well as the people involved. It also assists in identifying patterns and anomalies that may reveal other sources of evidence in a system’.

This step requires specifying a list of candidate definitions for all short-listed concepts (the definitions will be reconciled in Step 3.5). These are as follows: a Live acquisition is defined as ‘*a live data acquisition occurring when the system being analysed is still running while the analysis is being performed’*; a Collected Artefact is defined as ‘*The collected artefacts or information sources can hold malicious transactions to uncover any active unauthorized database access’;* and a Volatile artefact is defined as ‘*Volatile artefacts are collections of s related volatile database server and operating system artefacts (such as memories artefacts) which hold volatile data’*.

### 3.5 Reconciliation of definitions

Explicit definitions are important in science, as they improve communication and understanding. Precise definitions help to ensure that we are talking about the same phenomena. Further, they also assist in avoiding circular thinking where what appears as a thesis is only a redundant restatement of basic assumptions [99]. Differences between definitions are reconciled in this step. In choosing or synthesizing the common concept definition to be used, definitions shortlisted in Step 3.4 are considered.

The definitions have been developed by various people with varying backgrounds and perspectives. If there is a conflicting use of concept definition between two or more sources, then a process to reconcile and fit the definition is required. Some models neglect to explicitly define some of their concepts. In such cases, they do not provide any input to the reconciliation process. As an example, the concept of Data File is defined differently in five models as follows. Fowler [94] defines it as ‘a *data file where the database stores the events’*. Khanuja [3] defines it as ‘*data files attached to a trusted forensic machine and used to support activity reconstruction artefact analysis*’. Frühwirt [95] defines it as “*Databases store their content in so-called data files in the file system*”. Adedayo [96] defines it as “*The data files are used to store database objects such as tables and procedures”*. Fowler’s definition is too specific to database events. Khanuja’s is too specific to be able to use one of the database forensic investigation processes (analysis). However, Frühwirt’s and Adedayo’s definitions have somewhat the same meaning and purpose. Therefore, and to give a coherent and comprehensive definition, we choose Frühwirt’s and Adebayo’s definition as the basis of our generalized definition within our proposed concepts. In addition, we have added the keywords of other definitions to provide completeness of meaning. As a result, the Data File concept in our study is defined as “*The data files are used to store database objects and contents such as tables and procedures*. *Data files can also be attached to a trusted forensic machine and used to support activity reconstruction artefact analysis”* (See Table D in S4 Appendix II).

### 3.6 Designation of proposed common concepts into database forensic processes

Proposed concepts are designated into Database Forensic processes, specifically *Identification*, *Artefact Collection*, *Artefact Analysis*, *and Documentation & Presentation* [32]. *Identification* is a process in which Database Forensics identifies entire resources that may be used for investigative purposes. In addition, it identifies and detects database incidents. *Artefact Collection* is the process that collects and preserves data. It provides forensic technologies and artefacts by which to collect the evidence to effectively address database incidents. The *Artefact Analysis* process analyses collected evidence and reveal the causes of incidents; it also tracks the incidents and attacker. Lastly, *Documentation* & *presentation* will document the investigation processes and submit the report to the court. Designation into the processes is shown in Table 1.

**Table 1:** Concepts from step 3.7 designated into four DBF investigation processes.

Phase	Proposed Concepts
Identification	ForensicWorkstation, Company, Capture, UndoLog, LogFile, InvestigationTeam, Source, Artefact, VolatileArtefact, NonvolatileArtefact, DamagedDatabase, ModifiedDatabase, CompromisedDatabase, DatabaseAdministrator, Incident, DatabaseServer, DatabaseManagementSystem, IncidentResponding, LiveResponse, ForensicTechnique, Interview, Decision, Report
Artefact Collection	ForensicWorkstation, Source, Artefact, VolatileArtefact, NonvolatileArtefact, DatabaseFile, Logfile, UndoLog, Hashing, Backup, InvestigationTeam, ForensicTechnique, DataAcquisition, LiveAcquisition, DeadAcquisition, HybridAcquisition, DataCollected, Report, Integrity, OutputFile
Artefact Analysis	ForensicWorkstation,Reconstruction,Examination, TransactionLog, InvestigationTeam, ForensicTechnique, Evidence, IntruderActivity, MaliciousTransaction, Timeline, Report, DataCollected, DatabaseManagementSystem
Documentation & Presentation	InvestigationTeam, Evidence, Court, Source, Company

### 3.7 Identifying relationships between concepts and the resultant DBFM

**Fig 1:**
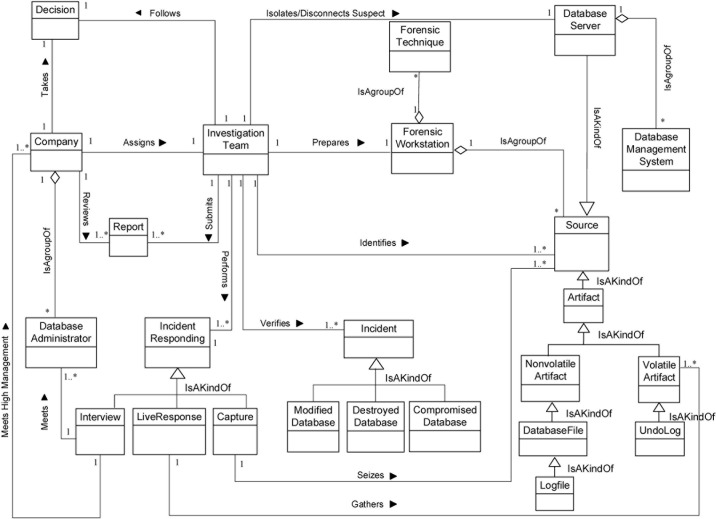
DBFM 1.0 identification-process class of concepts.

**Fig 2:**
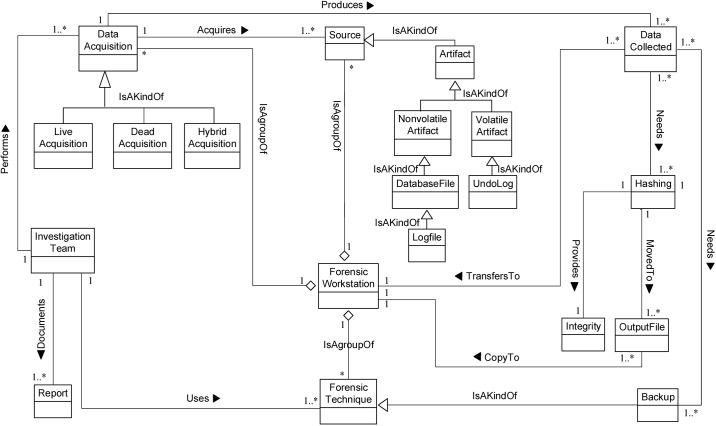
DBFM 1.0 artefact collection-process class of concepts.

**Fig 3:**
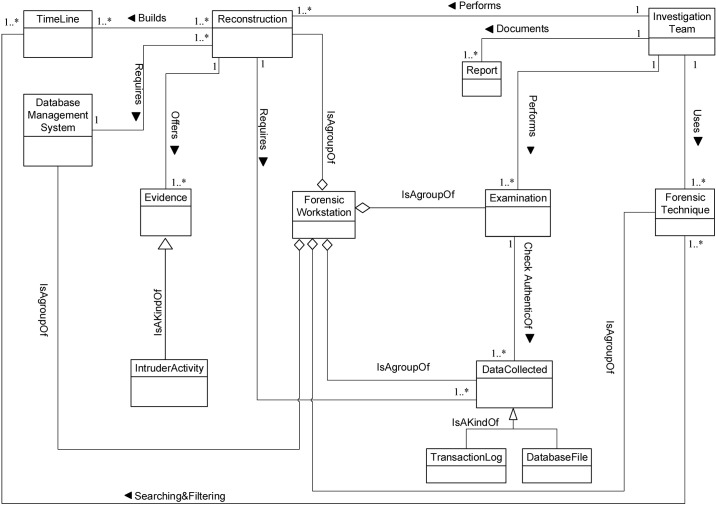
DBFM 1.0 artefact analysis process class of concepts.

**Fig 4:**
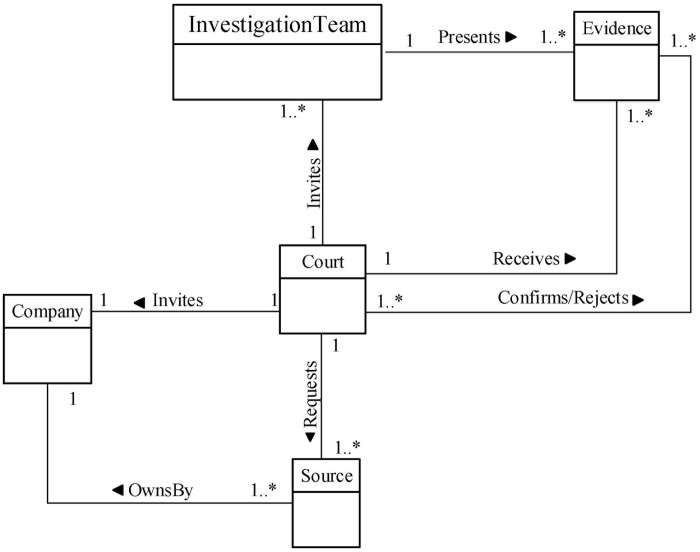
DBFM 1.0 documentation & presentation-process class of concepts.

We now determine the relationships between our Database Forensic Metamodel concepts. As shown in Figs 1–4, we use the (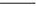), (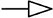) and (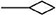) symbols to denote *Association*, *Specialization* and *Aggregation* relationships, respectively. As an association example, ‘*Verifies*’ between *InvestigationTeam* and *Incident* concepts indicates that an incident that could affect a company may need to be verified, revealed or detected. As specialization relationships, *VolatileArtefact* and *NonvolatileArtefact* specialize the *Artefact* concept. To use an aggregation example, *ForensicTechnique*, and *Source* are connected by the relation ‘*a grouping of*” during the identification, and artefact collection process. In almost all DBF models observed, we found the existence of *InvestigationTeam* during most of the Database Forensic models. More example of binary relationship is shown in Table 2. For each pair of a related concept, semantic of the relationship are identified and depicted with a specific symbol.

**Table 2:** Relationships among concepts in DBFM.

Concept 1	Relationship	Concept 2	Process/in Figure
InvestigationTeam	Association—‘Prepares’	ForensicWorkstation	Identification/1
InvestigationTeam	Association—‘Performs’	IncidentResponding	Identification/1
Interview	Association—‘Meets’	DatabaseAdministrator	Identification/1
Source	Aggregation—‘isAGroupOf’	ForensicWorkstation	Identification/1
DatabaseServer	Aggregation—‘isAGroupOf’	Source	Identification/1
VolatileArtefact	Specialization—‘IsAKindOf’	Artefact	Identification/1
TransactionLog	Specialization—‘IsAKindOf’	DataCollected	Artefact Analysis /3
DataAcquisition	Aggregation—‘isAGroupOf’	ForensicWorkstation	Artefact Collection/2
Source	Aggregation—‘isAGroupOf’	ForensicWorkstation	Artefact Collection /2
DataCollected	Association—‘Needs’	Hashing	Artefact Collection/2
DataCollected	Association—‘Needs’	Backup	Artefact Collection/2
Examination	Aggregation—‘isAGroupOf’	ForensicWorkstation	Artefact Analysis/3
VolatileArtefact	Specialization—‘IsAKindOf’	ArtefactAnalysis	Artefact Analysis/3
InvestigationTeam	Association—‘Performs’	Reconstruction	Artefact Analysis/3
Reconstruction	Association—‘Requires’	DataCollceted	Artefact Analysis/3
DatabaseFile	Specialization—‘IsAKindOf’	DataCollceted	Artefact Analysis/3
Court	Association—‘Invites’	Company	Documentation& Presentation/4
Court	Association—‘Receives’	Evidence	Documentation& Presentation/4
VolatileArtefact	Specialization—‘IsAKindOf’	Artefact	Identification and Artefact Collection (Inter processes) / 1 and 2
Source	Aggregation—‘isAGroupOf’	ForensicWorkstation	Identification to Artefact Collection (Inter phases) / 1 and 2

DBF is a continuous process with activities linking processes at different points. Correspondingly, in our DBFM, relationships between concepts are identified not only between concepts within the same process but also between concepts from different processes. Concepts from classes in different processes can be linked, and the continuous process in DBF can be formed. For example, *Investigator* (in the Identification process) is a concept designating an actor to formulate an investigation to meet future DBF needs based on extrapolations from the present needs. Investigation begins with the current status and charts out a path to a projected status, including short-term plans for achieving interim goals. Linkages across processes are established either through relationships between concepts from different processes or through common concepts between processes.

Linkages across processes are also established through common concepts between processes. The use of the concept *IncidentResponding* shows that the investigation task should start from the identification stage in any Database Forensic investigation process. Conversely, the use of the concept *ForensicTechnique* shows that the three processes require overlapping sets of *ForensicTechnique* for their process activities. Database Forensic Metamodel clearly presents classes of concepts in the four Database Forensic Metamodels as follows: *Identification*-process (Fig 1); *Artefact Collection*-process (Fig 2); *Artefact Analysis*-process (Fig 3); and *Documentation and Presentation*-process (Fig 4). The metamodel may also be used as a tool to determine the completeness of a given DBF solution.

### 3.8 Validation of Database Forensic Metamodel (DBFM)

The first version of DBFM will be validate**d** and improve**d** to make it complete and coherent. Thus, two common validation techniques will be used in this study, namely *Comparison against other models* and *Frequency-based selection*. Section 4.0 explains the process of DBFM validation in detail.

## 4.0 DBFM validation

We validate our DBFM for purposes of generality, expressiveness, and completeness. Validate generality ensures that the DBFM may cover whole DBF domain models, whereas validating the expressiveness ensures the degree to which it can directly model any particular real-world concept. This determines: that the theories and assumptions underlying the concepts in the metamodel are correct; and that the representation of the metamodel of the problem entity, the structure of the metamodel, and the logic and causal relationships are suitable for the intended purpose of the metamodel [17]. We apply two commonly used validation techniques as follows:

1. ***Comparison against other models***—Derived concepts of the developed metamodel are validated and compared to concepts from other similar (valid) existing domain models or metamodels [17,100,101]. For this purpose, we use a set of 10 Database Forensics models in a validation set (as listed in Table A in S1 Appendix I, and Table E in S5 Appendix III). We thoroughly ensured that every concept in each of the models can be appropriately derived from a concept within DBFM. Where required, we modified the DBFM to ensure that it can represent all models in the validation sets. This validation is described in sub-section 4.1, where we also list the changes we made to the first version of DBFM, yielding DBFM 1.1.
2. ***Frequency-based selection***—The importance of the individual concepts included in DBFM is evaluated as advocated in [102,103]. The second set of nine (9) models in Set V2 is used (see Table A in S1 Appendix I, and Table F in S6 Appendix III). This validation is described in sub-section 4.2, where we also list the changes we have made to DBFM 1.1, yielding DBFM 1.2.

### 4.1 DBFM validation 1—comparison against other models

**Fig 5:**
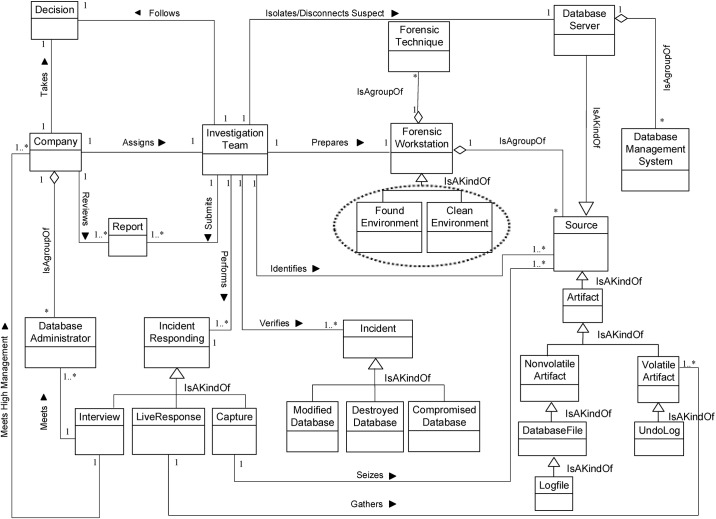
DBFM 1.1 a validated version of identification-process class of concepts.

**Fig 6:**
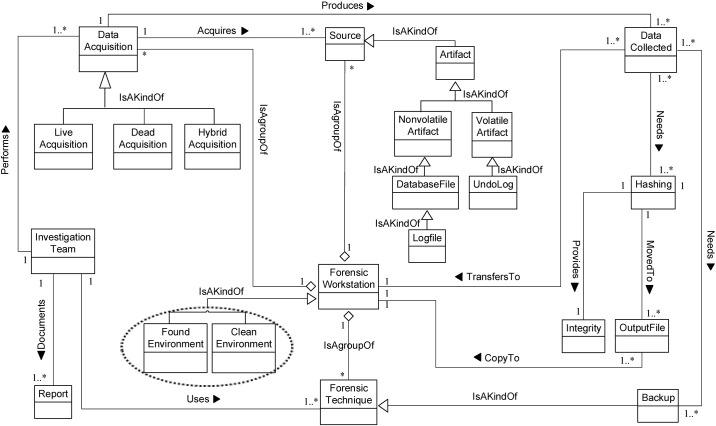
DBFM 1.1 a validated version of artefact collection-process class of concepts.

**Fig 7:**
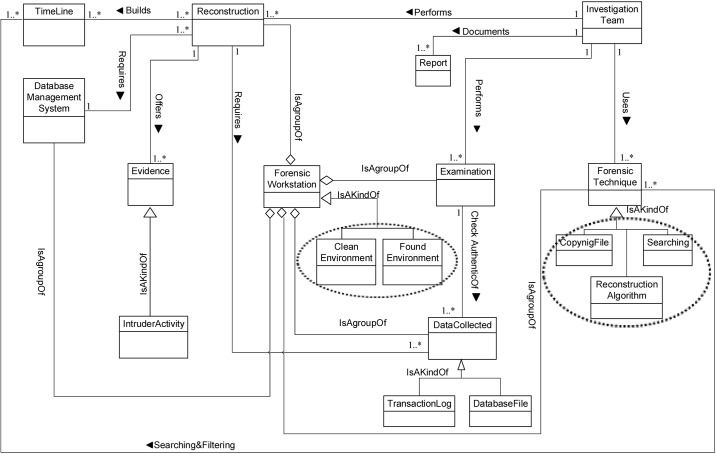
DBFM 1.1 a validated version of artefact analysis-process class of concepts.

**Fig 8:**
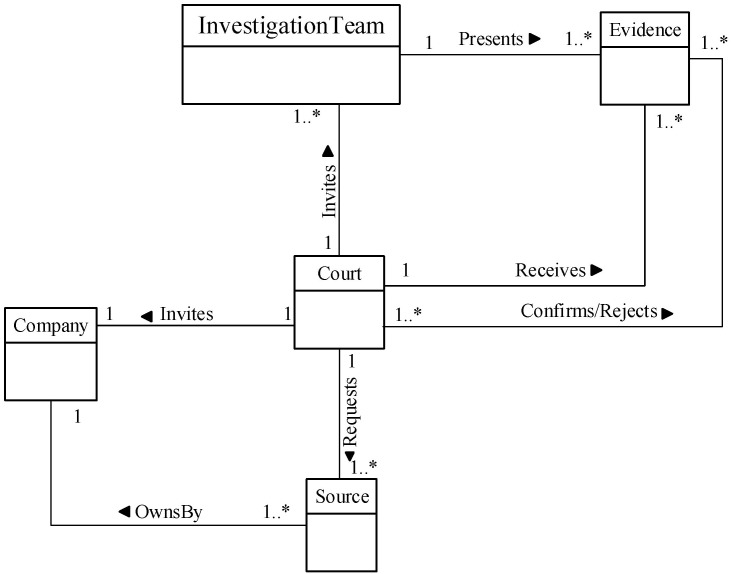
DBFM 1.1 a validated version of documentation & presentation-process class of concepts.

The first validation ensures that DBF can represent each of the models in the validation set V1 (as listed in Table A in S1 Appendix I, and Table E in S5 Appendix III). Where applicable, DBFM was modified to ensure that every model can be represented. DBFM was revised by adding 5 new concepts (listed in Table 3). Not all processes were changed to the same extent, e.g., Identification-process, Artefact Collection process and Artefact Analysis process of DBFM gained the *CleanEnvironment* and *FoundEnvironment* concepts as shown in Figs 5–7, whereas Documentation & Presentation-process added and changed relations among concepts (see Fig 8). The validation also confirmed the use of all relationships between all concepts (also shown in Table 4). None of the existing relationships were deleted.

**Table 3:** Five new added concepts based on validation over comparison to 10 models of set V1.

Concepts	Set V1	DBFM Phase	Concept Definition
ReconstructionAlgorithm	(5)	Analysis	Database reconstruction algorithm enables forensic investigators to determine whether data of interest was present in a database at an earlier time despite the fact that several database modifications may have been performed since that time.
Searching	(5)	Analysis	Searching each of the possible reconstructed relations before inserting it in the set relation.
CleanEnvironment	(5,7,9)	Identification, Collection, and Analysis	A clean environment is a setting where we have ensured that the data model will not alter the output of the DBMS. It is important to understand that a clean state differs from a post-mortem state characteristic of traditional digital forensics. A clean state is not merely a copy of the evidence that needs to be analyzed but rather a copy of the evidence program that runs like the original copy and from which we can query output. This means that the clean environment is set up to run like the original DBMS, but we are sure that the data model is not corrupting the output that we receive from the DBMS.
FoundEnvironment	(5,7,9)	Identification, Collection, and Analysis	A found environment refers to a state of the data model where the data model was in use in the DBMS when an event of forensic interest occurred. The found environment may also refer to an environment where the same machine that the DBMS was originally installed on is not used, but the data model was mirrored onto another machine. It is vital to understand that the found environment is not exactly the same here as the traditional meaning of a live digital forensic environment because the environment may fully or partially exist on the live machine or another machine.
CopyingFile	(9)	Analysis	Copy the data files from the suspect installation of the DBMS to a new installation of the DBMS, where a clean copy of the DBMS has been installed. The logic here is that a new installation of the DBMS on another machine will provide a clean data model. The data files of the new installation will be replaced with the data files of the suspect installation.

**Table 4:** List of relationships modifications between concepts in DBFM.

	Concept 1	Concept 2	Modification
**Process: Identification**			
1	CleanEnvironment	ForensicWorkstation	Add (Specialization)—isAKindOf
2	FoundEnvironment	ForensicWorkstation	Add (Specialization)—isAKindOf
**Process: Artefact Collection**			
1	CleanEnvironment	ForensicWorkstation	Add (Specialization)—isAKindOf
2	FoundEnvironment	ForensicWorkstation	Add (Specialization)—isAKindOf
**Process: Artefact Analysis**			
1	CleanEnvironment	ForensicWorkstation	Add (Specialization)—isAKindOf
2	FoundEnvironment	ForensicWorkstation	Add (Specialization)—isAKindOf
3	Searching	ForensicTechnique	Add (Specialization)—isAKindOf
5	CopyingFile	ForensicTechnique	Add (Specialization)—isAKindOf
6	ReconstructionAlgorithm	ForensicTechnique	Add (Specialization)—isAKindOf

### 4.2 DBFM validation 2—frequency-based selection

In this second validation, we perform a *Frequency-Based Selection* (*FBS*) technique using 9 models (Set V2 in Table A in S1 Appendix I, and Table F in S6 Appendix III). This is a *Feature Selection* technique that evaluates the importance of individual concepts in the model developed in [103]. It is based on the idea that the best model is formed using the most common features [104], and it is commonly used in data mining [105], software analysis [102], and medical retrieval systems [106]. By performing FBS, we remove *features* (*concepts*) that do *not have correlations to (or a need for)* the classification from DBFM.

**Fig 9:**
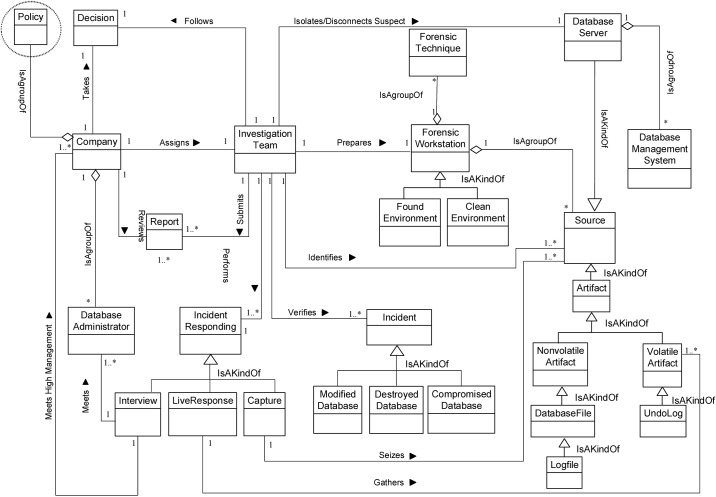
DBFM 1.2 A validated version of Identification-process class of concepts.

**Fig 10:**
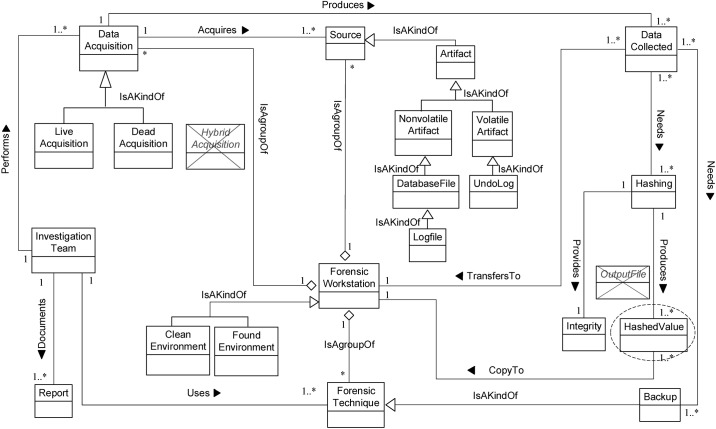
DBFM 1.2 a validated version of artefact collection-process class of concepts.

**Fig 11:**
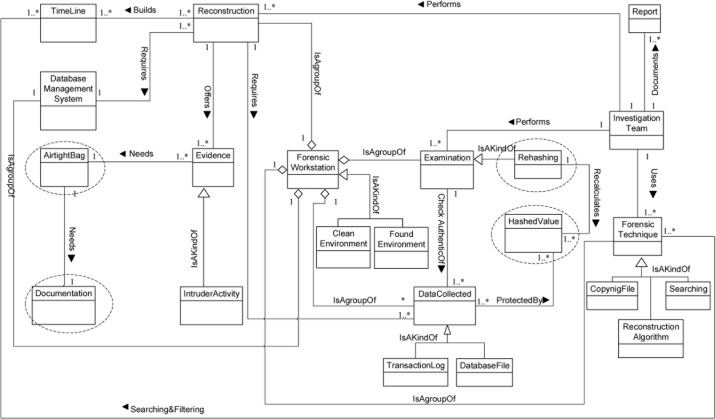
DBFM 1.2 a validated version of artefact analysis-process class of concepts.

**Fig 12:**
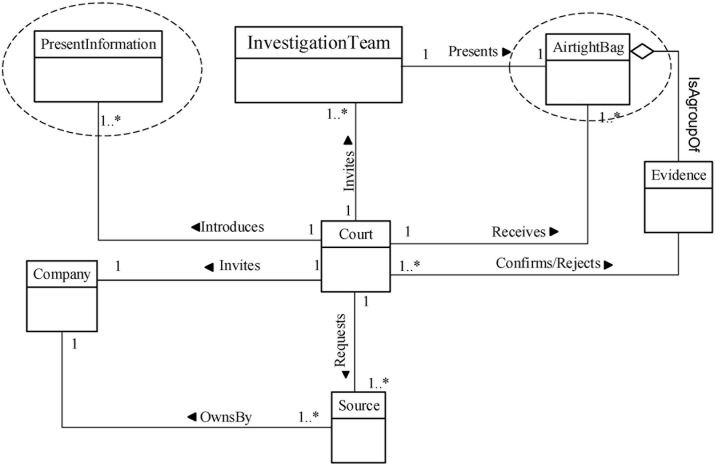
DBFM 1.2 a validated version of documentation & presentation-process class of concepts.

We first gather concepts from the models in the validation Set V2, and in doing so, we also ensure that they can all be refined using DBFM 1.1 (see Table F in S6 Appendix III). As expected, most concepts, in six of the nine models, were easily derived, and only the following six concepts were added to DBFM: *HashedValue*, *Rehashing*, *AirtightBag*, *PresentInformation*, *Documentation*, and *Policy* (see Figs 9–12). The second task in our FBS validation was to score each concept according to its frequency. Concepts that have a low score are revisited and are liable for deletion. The frequency results obtained for all DBFM concepts are shown in (Table G in S7 Appendix III, and Table H in S8 Appendix III).

Table 5 shows the result of this calculation for every DBFM concept. We have defined the following five categories of concepts based on their DoC values:

1. Very Strong (DoC result: 100–70%),
2. Strong (69–50%),
3. Moderate (49–30%),
4. Mild (29–11%), and
5. Very Mild (10–0%).

**Table 5:** Degree of confidence for DBFM concepts after FBS.

Percentage Degree (Degree of Confidence)				
100–70% (12 Very Strong)	69–50% (9 Strong)	49–30% (12 Moderate)	29–11% (13 Mild)	10–0% (3 Very Mild)
ForensicTechnique	ForensicWorkstation	Company	VolatileArtefact	UndoLog (√)
Source	InvestigationTeam	CleanEnvironment	DamagedDatabase	OutputFile (x)
Artefact	LogFile	FoundEnvironment	ModifiedDatabase	HybridAcquisition(x)
NonvolatileArtefact	TransactionLog	DatabaseFile	Interview	
Transaction	DatabaseServer	RedoLog	LiveResponse	
Incident	DataCollected	CompromisedDatabase	Capture	
DatabaseManagementSystem	CopyingFile	Report	Decision	
IncidentResponding	Examination	DataAcquisition	LiveAcquisition	
Integrity	DatabaseAdministrator	Hashing	DeadAcquisition	
Evidence		Backup	CollectedArtefact	
MaliciousTransaction		Reconstruction	TimeLine	
IntruderActivity		Court	Searching	
			ReconstructionAlgorithm	

Very Strong refers to the concept that appears many times in Set V2 models; whereas Very Mild is located at the other end of the scale. For example, the DBFM concept *Source* has a strong concept DoC value of 100% as follows:

$$\text{Doc }(Source)\;=\;\frac{8}{9}\text{ X }100\%\;=\;88$$

Aiming for absolute theoretical completeness is cited as a common bad practice in metamodel development [89]. As discussed in [89 pp. 23], similar to the development of domain-specific modelling, metamodel development is not about achieving perfection. We concur with these views, and if a DBFM concept has a ‘zero’ DoC score, it is deleted only after due consideration. Concepts with zero values are instead revisited and liable for deletion. DoC classification for all DBFM concepts is shown in Table 5 and described as follows: 12 concepts in DBFM 1.1 are categorized as ‘Very Strong’; 9 are “Strong’; 12 are ‘Moderate’; 13 are ‘Mild’; and 3 concepts are ‘Very Mild’ (Table 5).

The three very mild concepts are *UndoLog*, *OutputFile*, and *HybridAcquisition*, and they are reassessed in DBFM. We deleted *OutputFile* and *HybridAcquisition* as they only appeared rarely among domain models. However, we opted to keep *UndoLog* as they are common across varying DBF domains.

As a result of FBS, Figs 9–12 show the new validated version of Identification concepts, Collection concepts, Analysis concepts and Presentation concepts, respectively.

As a result of ensuring that DBFM represents each of the models in V2, we also added six concepts to the Identification process, Artefact Collection process, Artefact Analysis process and Documentation & Presentation process (encircled in (Figs 9–12)) as follows:

- *HashedValue*—“Values returned by hash functions”.
- *AirtightBag*—“where the evidence of CDROM should be kept in an environmentally neutral state”.
- *PresentInformation*—“Pictorial and graphical representation of statistics would help increase the understanding of people who needed to interface with the results”.
- *Policy*—“a set of ideas or a plan of what to do in particular situations that have been agreed to officially by a group of people, a business organization, a government, or a political party”.
- *Rehash*—“Matching is performed between old hash values with rehash tuple. If the hash value is the same, there is no problem”.
- *Documentation*—“clear details of names and roles of each person handling the bag of which there should not be many. The airtight evidence bag label should be impossible to get off the bag without breaking the seal which is unique to that bag”.

As a result of the refinements described in Sections 4.1 and 4.2, the DBFM classes of concepts are shown in (Figs 9–12). These figures have been annotated to clarify the impact of the FSB validation.

Practically, a real scenario has been utilized to assess the effectiveness and applicability of DBFM. This scenario was offered by [35]. “*A DBA believes that one of his development servers has been compromised*. *No auditing was enabled*. *Is there any evidence to support a compromise occurred”*. In this scenario where the company has no auditing services, we are going to demonstrate the capabilities of DBFM:

#### 1. DBFM identification class

The DBA of a company whose believes that one of his development servers has been compromised should notify the company management. The company looks at the nature, risk, type, and status of the incident. Then, if the incident is *critical*, the company should assign investigation team to identify, collect, preserve, analyze, reconstruct and document the evidence. The ***investigation Team*** should get authorization / search warrant to start their mission. Also, the access rights to reach the suspect database server should be there. Moreover, identify ***Sources*** which may use during the whole investigation task such as ***Artefact*, *Volatile Artefact*, *Non-volatile Artefact***, and ***Database server***. Artefact is a kind of sources which hold the evidence of incidents. Artefact has the volatile and non-volatile artefact. Volatile artefact includes database files, log files, database files, history files, trace files, alert files, transaction log, and so on. The non-volatile artefact includes the artefact which hold the volatile data such as undo log, SQL cash, log buffers, and whole memories structures.

The ***investigation Team*** should partially or completely isolate or disconnect the suspect the ***Database Server*** to get enough time to capture data. Additionally, the ***investigation Team*** should perform ***Incident Responding*** to check and verify the database incident. ***Incident Responding*** classified into three steps: **Capture** to seize and protect the **sources, Live Response** to gather the volatile artefact, and conduct an **interview** to get information.

***Investigation team*** should capture/seize the sources to protect it from tampering. Also, the most important step that must do by investigation team is a live response to gathering the live data from memory structure before missing.

Furthermore, investigation team should conduct an interview with IT staff such as DBA and also with the CEO of the company. The purpose of the interview with DBA is to get the information accounts, basic information about database, incident reports, network topology, database server activities, security procedures, and policies and so on. However, the purpose of the interview with CEO is to get information about the kind of privacy of the data of the company, and the importance of business continuous of the company and so on. Consequently, the *investigation team* should verify and check the type of incident and prepare and submit a primary investigation report to the company management. The company management takes the decision to stop or continues the investigation.

**Fig 13:**
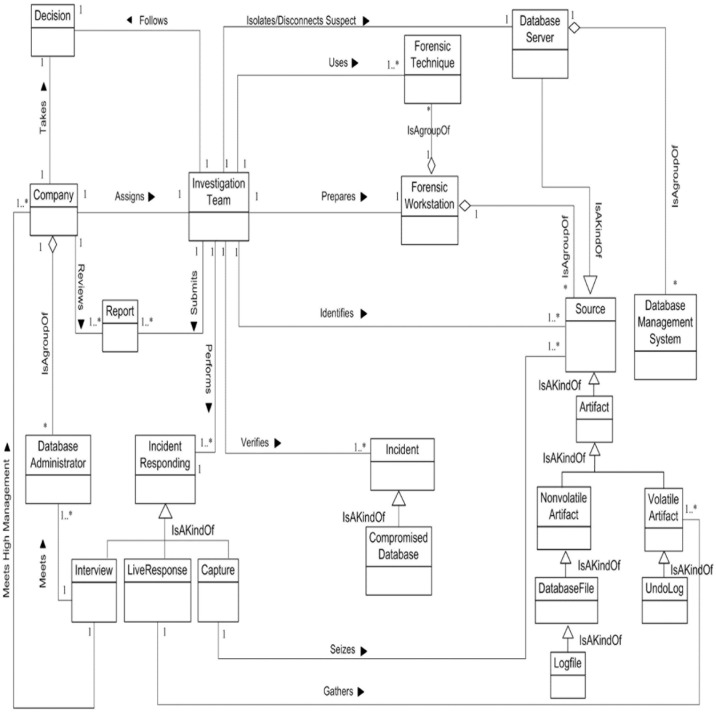
DBFM identification class of concepts.

Finally, the ***InvestigationTeam*** should prepare the ***ForensicWorkstation*** to achieve whole investigation task such collect, preserve analysis and document the evidence. The ***ForensicWorkstation*** is a clean and trust machine to do the investigation. It includes trust forensic techniques, trust and clean Database Management Systems (DBMS) to ensure the clean and correct results. ***Forensic Techniques*** is a collection of trusted forensic tools, methods, and algorithms that use by investigators to do an investigation. Fig 13 displays the identification metamodel class.

#### 2. DBFM artefact collection class

**Fig 14:**
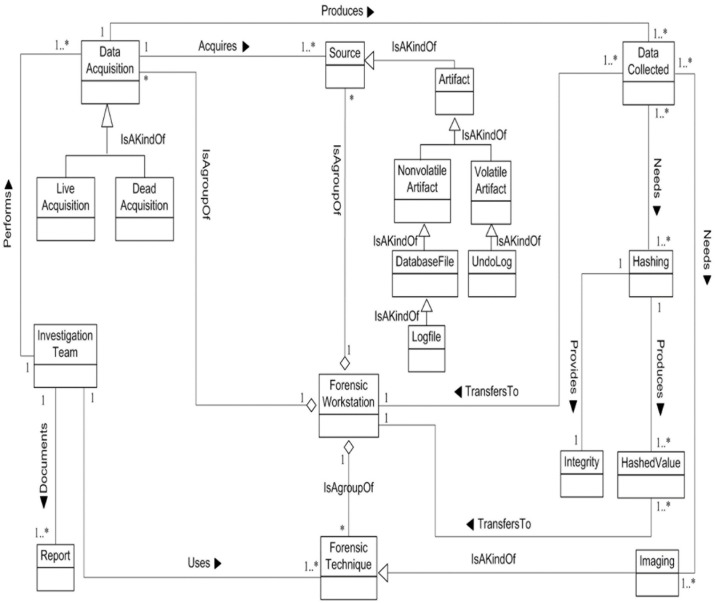
DBFM artefact collection class of concepts.

***Investigation Team*** has been already checked and identified the database incident, captured and seized the sources, identified and prepared the forensic workstation and forensic techniques in identification phase. Thus, in this phase, the volatile and non-volatile data should be acquired. ***Investigation Team*** performs ***Data Acquisition*** which acquires the different kinds of sources. ***Data Acquisition*** is a process which classified into the dead acquisition and lives acquisition. ***Dead Acquisition*** used to acquire the data from artefact when the database server is off, whereas the ***live acquisition*** is used to acquire the data from artefact when the database server is on. Furthermore, the investigation team should use special ***forensic techniques*** to acquire the data such as tools, algorithms, methods and so on. Consequently, the data collected will produce from data acquisition process. ***Data collected*** includes the volatile and non-volatile data. The data collected needs hashing algorithms and imaging to preserve the evidence from tampering. Hashing algorithms provide integrity of original data. The data collected and hashed value should keep and send to the ***forensic workstation***. Finally, investigation team should document all the collection and preservation steps in several ***reports***. Fig 14 displays Artefact collection metamodel class.

#### 3. DBFM artefact analysis class

Whole volatile and non-volatile data has been collected, preserved and moved to the forensic workstation in artefact collection phase. Thus, in this phase, the ***collected data*** should be examining, analyzing and reconstructing to reveal **any evidence to support a compromise occurred** and also determine who is tampering? When did tampering happen? Where did tampering happen?.

**Fig 15:**
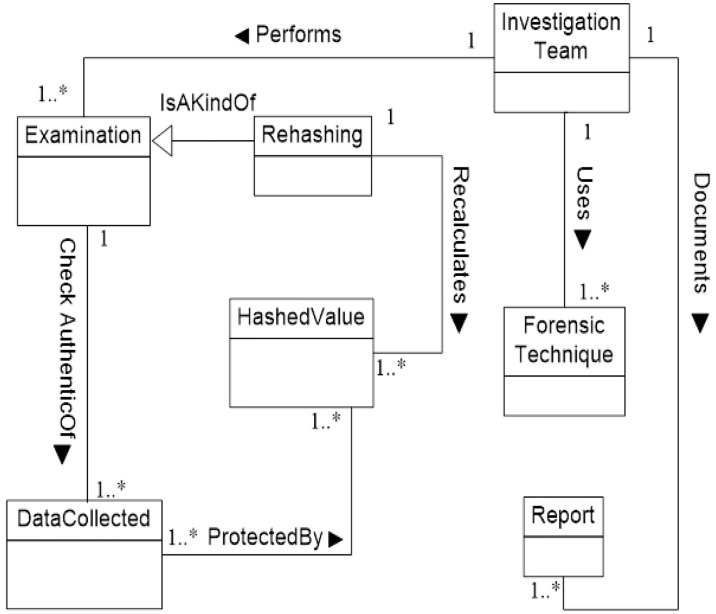
DBFM artefact analysis-process class of concepts.

First of all the ***collected data*** and ***hashed values*** that moved to ***forensic workstation*** should be examined to check the ***integrity*** of data. ***Investigation team*** performs ***examination process*** to check the authentic of collected data during moving / transforming to the forensic workstation. It includes ***rehashing*** of hashed value to ensure that the collected data has not tampered with. Furthermore, the investigation team documents the examination process in separate reports to introduce it in the court. However, if the results of examination process show that the ***collected data*** has been tampering with, then the ***investigation team*** needs doing collecting data again. Fig 15 displays examination process to check authentic of collected data.

**Fig 16:**
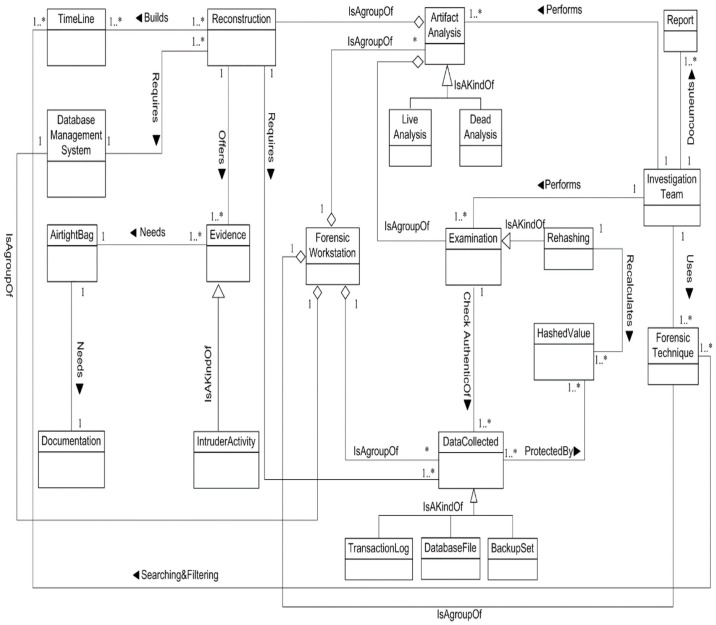
Documentation & presentation-process class of concepts.

Secondly, ***artefact*** ***analysis*** should perform by ***investigation team*** if the ***collocated data*** is cleaned and has not been tampering with. ***Artefact*** ***analysis*** includes reconstruction process. The ***reconstruction process*** is the cornerstone of the ***artefact*** ***analysis***. It includes constructing the ***timeline*** of events before or after the ***intruder actions***. The timeline events will be searching and filtering using ***forensic techniques*** to produce evidence. Basically, the ***investigation team*** will achieve ***reconstruction process*** to bring the evidence and detect whole tracks of the incident. ***Reconstruction process*** requires ***a collected data*** such as ***backup set*, *database files*, *and transaction log*** to build the timeline events. Also, it required a clean/exit ***database management systems*** (DBMS). The timeline events include whole events that done by intruder such as failure or success login/logoff, DML transactions, or DDL transactions etc. The investigator will use the special ***forensic techniques*** such as RGB/RGBY algorithms[57],Tile bitmap algorithm[46], or Log Miner [33], to search and filter the timeline events and detect who is tampering? When and where did tampering happen? The ***evidence*** that got by this process will organize, structure and preserve in **airtight bag/seal** to keep the evidence from temperature, humidity, and human tampering. The ***airtight bag*** should submit to the company and then the ***court of law***. However, all the artefact analysis process steps should be documented in several ***reports***. Fig 16 displays artefact analysis class.

Obviously, DBFM provides clear steps to guide a user’s, newcomers or practitioners to identify, collect, preserve, reconstruct and analyze database incidents. Therefore, users have the ability to instantiate a customized model to solve the problem in hand. For example, (Fig 16) depicts an examination model that derive from DBFM artefact analysis class.

## 5. Contribution

In this study, a *framework of a DBF language* underlined by a DBF metamodel (DBFM) is created. This demonstrates the emergence of a DBF semantic modeling standard through the metamodel that allows the description of various DBF data models. For the purpose of developing the DBFM, *general concepts* used in DBF are identified and new ones synthesized as required. This process involves analyzing the domain models, management processes, domain phases, activities, roles, goals and all other elements in DBF. The successful creation of the metamodel generalizes metamodelling to solve problems in DBF. The study generalizes the metamodelling approach. It creates a synthesis and validation processes that are discipline agnostic. In other words, the metamodelling process used in this study will not demand domain expertise as is usually the case in software engineering. Rather, the adapted process will provide new guidelines on sourcing the knowledge required to drive and validate the metamodelling process.

This study contributes to the facilitation of sharing of DBF knowledge. It presents a new a metamodeling based approach to guide DBF practitioners to structure, organize and reuse DBF-based on a Database Forensic Metamodel (DBFM). This is a specific artefact to describe a DBF language. The approach formulated does not mandate-consistent semantic relationships between the various stored models as it not fully formalized. Rather it is semi-formal and provides semantic links between the various models stored. The interpretation of the links is partly derived by the system users. This offers flexibility that enables easy storage of DBF knowledge by users and easy reuse by different users. From the metamodel, DBFM, semantic links between all models as well as new DBF models can be derived. DBFM is presented in four sets of concept classes: the *Identification*, *Artefact collection*, *Artefact analysis*, and Presentation & documentation class of concepts. Each set represents a corresponding DBF phase. This clearly describes the DBF domain to its users.

To the metamodeling community, the study contributes by facilitating the metamodeling process itself to not (knowledge) domain experts and prescribing a precise validation process that combines both domain and domain independent considerations. It first introduces new heuristics to a process adapted from [107] to enable preparing and selecting knowledge sources to drive the metamodeling process. This allows the domain metamodellers to be familiarized with common elements and various viewpoints of a domain without demanding deep DBF expertise. Improving the effectiveness of knowledge sources thus includes improving its usage and access. In addition, the study prescribes a precise validation using two types of metamodel validation techniques: *Comparison against other models*, *and Frequency-based Selection*. Each validation technique is tailored for a specific quality goal. The aim of the first validation, the *Comparison against other models* is to identify any missing concepts in the initial version of the DBFM and to also ensure its broad coverage. For the *Frequency-based Selection*, it aims to evaluate the importance of the individual concepts included in DBFM. As a result, a high degree of completeness, concepts significance and logical consistency of the metamodel are ensured.

Also, the study contributes to knowledge by also introducing a successful DBFM able to support different metadata types to define various DBF information models. It successfully describes the structure and functional description of any DBF information model as demanded from such a metamodel [108]. As the ability to offer modelling guideline to many domain users, DBFM helps various users to find rapid decision solution from semantic models earlier created. It successfully facilitates three meta-levels metamodeling abstraction in the MOF framework: the M2-level for a Metamodel, the M1-level for a Model, and the M0-level for a User Model.

DBFM through its four sets of classes (*Identification*, *Artefact collection*, *Artefact analysis* and Presentation & documentation classes) can give a picture of how all DBF actions should be executed. With better awareness and understanding of the whole DBF processes, many benefits can be derived because ensuring success in initial DBF phase will lead to success in the subsequent phases (solution models and the way they are arrived at from DBFM clearly vary according to the intention of the user for their new model. Finally, where coordination is lacking due to interoperability problems in a heterogeneous environment (e.g. different data formats or the absence of a common language), DBFM can also facilitate a general and global framework for coordinating people and data sources involved.

## 6. Limitations of the Model

Our Database Forensic metamodel (DBFM) has been developed based on a careful analysis of the existing literature and domain-specific database forensic models. It has been validated through a couple of iterations and applied to a specific case. While the DBFM is generic and domain-independent and can be instantiated for specific DBF scenarios, it has some limitations that need to be addressed, especially as the DBFM evolves.

The following are some of the limitations that will be taken into account in the next iteration of DBFM refinement.

- In developing our DBFM, we have considered only the models presented in English that could lead to a cultural bias. In the next stage of our model refinement, we will consider a more diverse set of models from different geographic regions, which will improve the completeness and applicability of the model.
- While we searched for input from practitioners and experts during the DBFM development, we validated the model against two sets of models from the literature and applied it to a specific DBF case. There was no formal evaluation of the model performed by the experts in Database Forensics. As part of a future work, the DBFM and the results will be validated by a group of DBF experts or digital forensic experts.
- In the second part of the DBFM validation process (Section 4.2), we used a frequency-based selection technique. In this process, we were interested in knowing whether a particular concept appeared in a model or not and did not focus on how many times it appeared. In the field of information retrieval, this frequency is used to indicate the importance of a concept. In the next iteration of DBFM development, we will consider this fact and refine the model accordingly.
- In developing the DBFM, our goal was to create a model that is general and complete. Hence, we tried to include all the concepts that occurred in all or most of the models. While this resulted in a broad model that represents common practice, it might not be the “best practice” model. Our DBFM might be read as a model that is useful for training novices but may not be useful in helping organizations develop a state-of-the-art or best-practice model. While specialized (best-practice) models can be instantiated from the general DBFM, in our future work, we will take a synthesis approach and incorporate most important concepts from the various models to produce a class of best-practice models.
- As the DBFM is designed to support DBFM knowledge reuse, the ultimate test of DBFM would be in the deployment of a knowledge repository developed using DBFM. In other words, it operates using a DBFM-based repository to support DBF business processes. This will first test the expressivity of DBFM through interactions with domain experts from different databases and the reusability of the stored knowledge. For instance, in this work, we assume that DBF knowledge is symbolic (similar to other existing models). Whether this assumption will hinder the applicability of the approach, and to what extent, can only be assessed empirically once a knowledge repository is deployed using the metamodel.

## 7. Conclusion

This study has discussed the development of the Database Forensic Metamodel (DBFM). The metamodel presented is intended to become an effective platform for sharing and integrating DBF knowledge from varying sources. Existing database forensic models are not based on any metamodels or standards but rather constitute proprietary solutions that are mainly focused on frameworks and other model aspects. This is the first work that develops a DBF metamodel across the four established phases of the database forensic domains. Our DBFM can unify these works as a navigation metamodel. More importantly, the DBFM is the first step to allow interoperability of DBF solutions and effective transfer of knowledge across database boundaries. It may also be used as a tool to determine the completeness of any DBF solutions.

We presented the metamodel in a familiar format, UML, to increase its ease of use and broaden its appeal. In synthesizing our metamodel, we used 18 models for the development of DBFM. The future work of this study will concentrate on development of a database forensic repository to demonstrate the effectiveness of DBFM.

## Supporting information

S1 Appendix I Table A. The 38 DBF models for development (Set I) and validation (Set II). The perspectives they cover are denoted by ‘√’.
(DOCX)

S2 Appendix I Table B. Extracted database forensic concepts. (DOCX)

S3 Appendix I Table C. Candidate and Proposed common concepts for the DBF domain. (DOCX)

S4 Appendix II Table D. List of proposed DBF concepts and definition. (DOCX)

S5 Appendix III Table E. Validation summary against model set V1. (DOCX)

S6 Appendix III Table F. Validation summary against model set V2. (DOCX)

S7 Appendix III Table G. Frequency results of identification and artefact collection-process concepts. (DOCX)

S8 Appendix III Table H. Frequency result of artefact analysis and documentation & presentation-process concepts. (DOCX)
