# Supplementary material for: Development and validation of a Database Forensic Metamodel (DBFM)
Source: PLoS One. 2017 Feb 1;12(2):e0170793. doi: 10.1371/journal.pone.0170793 (PMC5287479; doi:10.1371/journal.pone.0170793)
Supplement: S1 Appendix I — The perspectives they cover are denoted by ‘√’. (DOCX) [file pone.0170793.s001.docx]

**S1 AppendixI.Table A. The 38 DBF models for development (Set I) and validation (Set II). Perspectives they cover are denoted by ‘√’.**

| **SET I (To be used to develop the initial DBFM)** | | **Year** | **Database**  **Dimensions** | **Database Forensic**  **Technology** | **Database Forensic**  **Investigation process** |
| --- | --- | --- | --- | --- | --- |
|  | System and method for investigating a data operation performed on a database [[20](#_ENREF_20)] | 2004 | √ | √ | √ |
|  | Forensic Analysis of a SQL Server 2005 Database Server [[42](#_ENREF_42)] | 2007 | √ | √ | √ |
|  | Oracle Forensics Live Response [[22](#_ENREF_22)] | 2007 | √ | √ | √ |
|  | SQL Server Forensic Analysis Methodology [[19](#_ENREF_19)] | 2008 | √ | √ | √ |
|  | Database forensic investigation based on table relationship analysis techniques [[43](#_ENREF_43)] | 2009 | √ | √ | √ |
|  | Evidence of Investigation Methodologies for Detecting Financial Fraud based on Forensic Accounting [[44](#_ENREF_44)] | 2009 | √ | √ | √ |
|  | Metadata context in Database Forensics [[1](#_ENREF_1)] | 2009 | √ | √ |  |
|  | The Method of Database Server Detection and Investigation in the Enterprise Environment [[55](#_ENREF_55)] | 2011 | √ | √ | √ |
|  | Digital Evidence for Database Tamper Detection [[56](#_ENREF_56)] | 2012 | √ | √ | √ |
|  | Framework for Database Forensic Analysis [[2](#_ENREF_2)] | 2012 | √ | √ | √ |
|  | A Workflow to Support Forensic Database Analysis [[57](#_ENREF_57)] | 2012 | √ | √ | √ |
|  | Dimensions of Reconstruction in Database Forensic [[24](#_ENREF_24)] | 2012 | √ | √ | √ |
|  | Forensic Analysis of Databases by Combining Multiple Areas of Evidence [[23](#_ENREF_23)] | 2013 | √ | √ | √ |
|  | Database Forensic Investigating Compromised Database Management Systems [[58](#_ENREF_58)] | 2014 | √ | √ | √ |
|  | Role of metadata in forensic analysis of database attacks [[40](#_ENREF_40)] | 2014 | √ | √ | √ |
|  | Towards a forensic-aware database solution: Using a secured database replication protocol and transaction management for digital investigations [[38](#_ENREF_38)] | 2014 |  | √ | √ |
|  | Ideal log setting for database forensics reconstruction [[39](#_ENREF_39)] | 2015 | √ | √ | √ |
|  | Database Forensic analysis through internal structure carving [[41](#_ENREF_41)] | 2015 | √ | √ | √ |
| **SET V1 (To be used for first validation)** | | | | | |
| 1 | Forensic tamper detection in SQL server [[59](#_ENREF_59)] | 2006 |  | √ | √ |
| 2 | Finding Evidence of Data Theft in the Absence of Auditing [[60](#_ENREF_60)] | 2007 |  | √ | √ |
| 3 | Discovering Methodology and Scenario to Detect Covert Database System [[61](#_ENREF_61)] | 2007 |  | √ |  |
| 4 | RECONSTRUCTION IN DATABASE FORENSICS [[62](#_ENREF_62)] | 2012 | √ |  |  |
| 5 | Enriching Forensic Analysis process for Tampered Data in Database [[63](#_ENREF_63)] | 2012 |  |  | √ |
| 6 | Arguments and Methods for Database Data Model Forensics [[64](#_ENREF_64)] | 2012 | √ | √ |  |
| 7 | InnoDB database forensics: Enhanced reconstruction of data; manipulation of queries from redo logs [[65](#_ENREF_65)] | 2013 |  |  | √ |
| 8 | An Improved Framework for Tamper Detection in Databases [[67](#_ENREF_67)] | 2015 |  | √ | √ |
| **SET V2 (To be used for Second validation)** | | | | | |
| 1 | Oracle database forensics using LogMiner [[68](#_ENREF_68)] | 2004 | √ | √ |  |
| 2 | Efficient model for detection data and data scheme tempering with purpose of valid forensic analysis [[69](#_ENREF_69)] | 2009 | √ | √ | √ |
| 3 | Methods for Efficient Digital Evidence of Collection of Business Processes and Users’ Activity in eLearning Environments[[70](#_ENREF_70)] | 2010 |  | √ | √ |
| 4 | Detecting Database Attacks Using Computer Forensics Tools  [[71](#_ENREF_71)] | 2011 |  | √ | √ |
| 5 | An approach to examine the Metadata and Data of a Database Management System by making use of a forensic comparison tool [[72](#_ENREF_72)] | 2011 | √ | √ | √ |
| 6 | A framework for discovering internal financial fraud using analytics [[73](#_ENREF_73)] | 2011 | √ | √ | √ |
| 7 | Combining Digital Forensic Practices and Database Analysis as an Anti-Money-Laundering Strategy for Financial Institutions [[74](#_ENREF_74)] | 2012 |  | √ | √ |
| 8 | Database Tampering and Detection of Data Fraud by using the Forensic Scrutiny Technique [[75](#_ENREF_75)] | 2012 | √ | √ |  |
| 9 | Forensic Investigation of MySQL Database Management System [[76](#_ENREF_76)] | 2014 |  | √ | √ |
