# Supplementary material for: Development and validation of a Database Forensic Metamodel (DBFM)
Source: PLoS One. 2017 Feb 1;12(2):e0170793. doi: 10.1371/journal.pone.0170793 (PMC5287479; doi:10.1371/journal.pone.0170793)
Supplement: S2 Appendix I — (DOCX) [file pone.0170793.s002.docx]

**S2 AppendixI.Table B. Extracted Database Forensic Concepts.**

| **Models** | **Extracted Concepts** | **Total** |
| --- | --- | --- |
|  | Capture evidence, Intruder activity, Database server, Collected data, Reconstructing database, Redo log, Undo log, Audit trail, Alert log, Hashing, Examination, Backup, Transaction, Investigation Team, Resources, Damage Database*.* | 16 |
|  | Data acquisition, Database server, Data file, Capture, Acquired data, Transaction logs, Evidence integrity, Suspicious transaction, Evidence, Volatile data, Non-volatile data, Event, Output file, Log file, Database files, Incident response, Trusted forensic workstation, and Timeline. | 18 |
|  | Redo log, Gathering evidence, Hashing, Examination, Forensic examiner, Suspicious activity, Volatile evidence, Non-volatile evidence, Incident, Database Server, Output file, Organization, Court, Data file, Live response, Collection server, Decision | 17 |
|  | Live acquisition, Dead acquisition, Hybrid acquisition, Database server, Collected data, Reconstruction, Investigation team, Event, DBMS, Incident responding, Organization, Forensic Workstation, Data file Volatile artefact, Non-volatile Artefacts | 15 |
|  | Database server, Capture, Data collected, Monitoring, Investigation team, DBMS, Company, Investigation extraction methods | 8 |
|  | Data seizure, Data acquisition, Seizure , Data collected, Examiner, Data resources, Volatile evidence, Company, Court, Interview, Report | 11 |
|  | Examination, Backup, Investigation Team, Source, Evidence, Incident, DBMS, Court, Capture | 9 |
|  | Data collection, Database server, Data collected, Database Server, Output file, Database files, Company, Investigator computer, Interview | 9 |
|  | Redo log, Database server, Examination, Investigation Team, Evidence, Volatile evidence, Non-volatile evidence, Event, Database Server, Log file, Organization, Live response, Evidence collection server, Evidence location, Timeline | 15 |
|  | Redo log, Undo log, Database Activity, Database server, Collected data, Reconstruction, Backup, Transaction, Investigation team, Resources, Evidence, Database intrusion, Event, Database Server, Log file, Data file, Court of law, Trusted forensic machine, Timeline, Forensic report, Artefact | 21 |
|  | Capture, Data collected, Auditing, Examination, Live analysis, Evidence, Volatile data, Non-volatile evidence, Database administrator, Decision, Artefact | 11 |
|  | Live acquisition, Dead acquisition, Hybrid acquisition, Data acquisition, Data collection, Database server, Collected data, Reconstruction, Transaction logs, Investigation team, Source, Damaged database, Modified Database, Compromised database, Database Forensic Tool, Database log file, Data file, DBMS, Live response, Volatile artefact, Non-volatile Artefacts | 21 |
|  | Intruder transactions, Transaction, Investigation team, Source, Organization, Malicious transactions, Artefact | 7 |
|  | Hashing, Rebuilding, Backup, Forensic investigator, Evidence Integrity, Source, Modified Database, Compromised database, Incident, Database Forensic Tools, Log file, Company, Forensic environment, Data file, Forensic techniques | 16 |
|  | Reconstruction event, Transaction logs, Investigation team, Source, Evidence, Database administrator, Log file, Database files, Court of law, Malicious transactions, Final forensic report | 11 |
|  | Redo log, Undo log, Data collected, Reconstruction, Hashing, Backup, Transaction, Vital source, Database administrator, Log file, Data file, DBMS, Court, Timeline | 14 |
|  | Redo log, Reconstruction, Transaction logs, Investigation team, Incident, Log file, Data files, Timeline | 8 |
|  | Reconstructing, Rebuilding data structures, Transaction, Volatile data, Database administrator, Database files, DBMS | 7 |
