# Supplementary material for: Development and validation of a Database Forensic Metamodel (DBFM)
Source: PLoS One. 2017 Feb 1;12(2):e0170793. doi: 10.1371/journal.pone.0170793 (PMC5287479; doi:10.1371/journal.pone.0170793)
Supplement: S3 Appendix I — (DOCX) [file pone.0170793.s003.docx]

**S3 AppendixI.Table C. Candidate and Propose a common concepts for DBF domain.**

| **No** | **Propose Common Concepts** | **Candidate Concepts** | **Frequency** | **Generality** | **Definition** |
| --- | --- | --- | --- | --- | --- |
|  | Capture | Capture | 3 | 1 | 1 |
|  |  | Seizure | 1 | 1 | 1 |
|  | Data acquisition | Gathering evidence | 1 | 1 | 1 |
|  |  | Data acquisition | 3 | 1 | 1 |
|  | Intruder activity | Intruders transactions | 1 | 1 | 0 |
|  |  | Intruder activity | 2 | 1 | 1 |
|  | Data collected | Collected data | 3 | 1 | 1 |
|  |  | Data collected | 5 | 1 | 1 |
|  |  | Acquired data | 1 | 1 | 1 |
|  | Reconstruction | Reconstructing database | 1 | 1 | 1 |
|  |  | Reconstruction | 5 | 1 | 1 |
|  |  | Reconstruction event | 1 | 1 | 1 |
|  |  | Reconstructing | 1 | 1 | 0 |
|  | Redo log | Redo log | 6 | 1 | 1 |
|  | Undo log | Undo log | 3 | 1 | 1 |
|  | Hashing | Hashing | 4 | 1 | 1 |
|  | Examination | Examination | 5 | 1 | 1 |
|  | Backup | Backup | 5 | 1 | 1 |
|  | Transaction | Transaction | 4 | 1 | 1 |
|  | Transaction logs | Transaction logs | 4 | 1 | 1 |
|  | Investigation Team | Investigation Team | 9 | 1 | 1 |
|  |  | Forensic examiner | 1 | 1 | 1 |
|  |  | Examiner | 1 | 1 | 1 |
|  | Integrity | Evidence integrity | 1 | 1 | 1 |
|  |  | Integrity | 2 | 1 | 1 |
|  | Source | Resources | 1 | 1 | 1 |
|  |  | Source | 5 | 1 | 1 |
|  | Evidence | Evidence | 6 | 1 | 1 |
|  | Damaged database | Damaged database | 2 | 1 | 1 |
|  | Modified Database | Modified Database | 2 | 1 | 1 |
|  | Compromised database | Compromised database | 2 | 1 | 1 |
|  | Database administrator | Database administrator | 4 | 1 | 1 |
|  | Incident | Event | 4 | 1 | 1 |
|  |  | Incident | 5 | 1 | 1 |
|  | Database Server | Database Server | 10 | 1 | 1 |
|  | Output file | Output file | 3 | 1 | 1 |
|  | Log files | Log file | 8 | 1 | 1 |
|  |  | database log file | 1 | 1 | 0 |
|  | Database files | Database files | 5 | 1 | 1 |
|  | Data file | Data file | 8 | 1 | 1 |
|  | DBMS | DBMS | 7 | 1 | 1 |
|  | Incident responding | Incident response | 1 | 1 | 0 |
|  |  | Incident responding | 1 | 1 | 1 |
|  | Company | Organization | 4 | 1 | 1 |
|  |  | Company | 5 | 1 | 1 |
|  | Court | Court | 5 | 1 | 0 |
|  |  | Court of law | 2 | 1 | 0 |
|  | Live response | Live response | 3 | 1 | 1 |
|  | Forensic workstation | Forensic environment | 1 | 1 | 1 |
|  |  | Forensic workstation | 2 | 1 | 1 |
|  |  | Evidence collection server | 1 | 1 | 0 |
|  |  | collection server | 1 | 1 | 1 |
|  |  | Investigator computer | 1 | 1 | 1 |
|  | Forensic Technique | Forensic Techniques | 2 | 1 | 1 |
|  |  | Investigation extraction methods | 1 | 1 | 1 |
|  | Malicious transaction | Malicious transactions | 2 | 1 | 1 |
|  | Timeline | Timeline | 5 | 1 | 1 |
|  | Interview | Interview | 2 | 1 | 1 |
|  | Volatile artefact | Volatile artefact | 2 | 1 | 1 |
|  | Non-volatile Artefact | Non-volatile Artefact | 2 | 1 | 1 |
|  | Decision | Decision | 2 | 1 | 1 |
|  | Report | Forensic report | 1 | 1 | 0 |
|  |  | Report | 2 | 1 | 1 |
|  |  | Final forensic report | 1 | 1 | 0 |
|  | Artefact | Artefacts | 3 | 1 | 1 |
|  | Live Acquisition | live acquisition | 2 | 1 | 1 |
|  | Dead Acquisition | Dead acquisition | 2 | 1 | 1 |
|  | Hybrid Acquisition | Hybrid acquisition | 2 | 1 | 0 |
