# Supplementary material for: Development and validation of a Database Forensic Metamodel (DBFM)
Source: PLoS One. 2017 Feb 1;12(2):e0170793. doi: 10.1371/journal.pone.0170793 (PMC5287479; doi:10.1371/journal.pone.0170793)
Supplement: S4 Appendix II — (DOCX) [file pone.0170793.s004.docx]

**S4 AppendixII.Table D. List of Proposed DBF Concepts and Definition.**

| **No** | **Common Concept** | **Source Definition** | **Reconciled Definition** |
| --- | --- | --- | --- |
|  | Capture | 1. M2: (Capture) – capture related information via customized windows forensic tool during verification stage of investigation. 2. M5: (Capture) – capture query packet and analyses inner join and outer join of the query, and then extracts relationship. 3. M6: (Seizure) – extract fraud data from the server. 4. M7: (Capture) – capture data that has been (partially) destroyed, or only partially recovered. 5. M11: (Capture) – capture database related incident either by user reported events and/ or system audit. | Capture process is capturing related database information/data via customized windows forensic tool, user reported events or system audit during verification stage of investigation that has been partially destroyed, or only partially recovered from server |
|  | Data acquisition | 1. M3: (Gathering evidence) – The evidence which is gathering by incident responder of the attack as it happens. 2. M2: (Data acquisition) – null; 3. M6: (Data acquisition) – Extract evidence that relates to account fraud. 4. M12: (Data acquisition) – acquisition of data from a modified database that has not been damaged or compromised. | Data acquisition is gathering/ acquiring evidence data that relates to account fraud from a modified database that has not been damaged or compromised by incident responder of the attack as it happens. |
|  | Intruder activity | 1. M1 :( Intruder activity) – Intruder activities are the activities which done by intruder to harm the confidentially and integrity of database. 2. M12: (Intruder activity) – activities that determined during analysis collected data; 3. M13: (Intruders transactions) –null | Intruder activities are the activities which done by intruder to harm the confidentially and integrity of database. These activities determined during analysis collected data. |
|  | Data collected | 1. M1: (Collected data) – are various data relating to database activity and the status of database contents which include evidence of what the intruder did, and metadata regarding the intruder’s activity. 2. M2: (Acquired data) – acquired physical log files using the dcfldd disk imaging tool. 3. M5: (Data collected) – can be used for the investigation with information of the file server related to the database. 4. M6: (Data collected) – can be used for the investigation with information of the file server related to the database. 5. M8: (Data collected) – the data which collected during the collection phase. 6. M10: (Collected data) - collected data is a data that submitted as evidence in a court of law. 7. M11: (Data collected) – data collected is formulated into a report and a final decision is made to either proceed with the examination or document the reason for the trigger and close the case. 8. M12: (Collected data) – the data which collected for analysis. 9. M17: (Data collected) – the data that collected by the rollback mechanism. | Data collected are a data that collected during collection process that can be used for the investigation/analysis process. It includes various data relating to database activity, physical log files, and file server related to the database. Furthermore, these data include evidences of what the intruder did and metadata regarding the intruder’s activity. |
|  | Reconstruction | 1. M1:(Reconstructing database) - Reconstructing the database may entail restoring logs in addition to undo and/or redo logs, replaying contents of an SQL cache and replaying individual queries and/or transactions. 2. M4: (Reconstruction) – activity reconstruction involves retracing past system and user database activity that identifying the commands executed by the attacker on the SQL Server during his or her period of unauthorized access. 3. M10: (Reconstruction) – information can be used to reconstruct past SQL execution history resulting from ad hoc queries, stored procedures, or function execution. 4. M12: (Reconstruction) – reconstruction may involve the recovery of data from proprietary formats. 5. M17: (Reconstruction) –reconstruction of the sequence of changes. 6. M18: (Reconstruction) - reconstruction of data that might have existed on a database at earlier time prior to some modifications or deletion. 7. M16: (Reconstruction event) - the reconstruction of events and activities from the collected volatile and non-volatile artefacts is done for validation against the previously generated log analysis report to give a final forensic report. At this stage different database attacks like Sql injection, brute force, buffer overflow, and reconnaissance attacks will be detected on one side along with major anti-forensics attacks like trail obfuscation, artefact wiping on the other. Database log information will be recovered in case it is modified by any of the attacks. The evidence will consist of who, why, when, what, how and where the malicious transaction were carried out. 8. M19: (Reconstructing) – reconstructing the fact that data was inserted, deleted or updated | Reconstruction is a process to reconstruct events that might be responsible for an incident from collected volatile and non-volatile artefacts/data which involve retracing past system, user database activity, past SQL execution history, stored procedures, and function execution. Also, database log information will be recovered in case it is modified by any of the attacks. |
|  | Redo log | 1. M1: (Redo log) –capture all data changes and may also capture other activities. 2. M3: (Redo log) –used in locating dropped objects and recovering data in an Oracle database; 3. M9: (Redo log) – are the current changes that have not been check pointed into the data files. 4. M10: (Redo log) – The redo log stores the information which is utilized at the time of crash recovery process. It gives permission to the recovery process so that the transactions are re-executed that could or could not have completed before the crash. 5. M17: (Redo log) – The purpose of redo logs in general is to apply changes that were made in the memory, but where not flushed to the permanent table records. They are used in order to recover the database from an inconsistent state provoked by a crash. 6. M18: (Redo log) – The redo log in Oracle is the most important part of Oracle when it comes to recovery or forensics analysis. | The redo log stores the information which is utilized at the time of crash recovery process. It gives permission to the recovery process so that the transactions are re-executed that could or could not have completed before the crash. The purpose of redo logs in general is to apply changes that were made in the memory, but where not flushed to the permanent table records. They are used in order to recover the database from an inconsistent state provoked by a crash |
|  | Undo log | 1. M1: (Undo log) – Allow an investigator to replay or reverse a series of changes involving any number of cells in a table, which may allow him or her to closely track the intruder’s actions. 2. M10: (Undo log) – The undo log allow rolls back transactions. It also displays the older versions of the data. 3. M17: (Undo log) - Undo logs are used in case a user does not complete a transaction with a commit or rollback to undo the previously executed actions. | Undo logs are used in case a user does not complete a transaction with a commit or rollback to undo the previously executed actions. It may allow an investigator to replay or reverse a series of changes involving any number of cells in a table, which may allow him or her to closely track the intruder’s actions. |
|  | Hashing | 1. M1: (Hashing) – the hash may be used to ensure that the database forensics techniques that will be applied have not changed the data, by comparing the hash to a hash generated at another time. 2. M3: (Hashing) –hash has been created using the sum of every line of source once it has been hashed. These hashes can then be compared against a list of known hashes. 3. M14: (Hashing) – hash can be calculated on the files of a new clean data model installation and then compared to the md5 of the files on the found data model. 4. M17: (Hashing) - assure consistency of transmitted data between the master and the slaves, data integrity and authenticity must be assured. | The hash may be used to ensure that the database forensics techniques that will be applied have not changed the data, by comparing the hash to a hash generated at another time. The consistency of transmitted data between the master and the slaves, data integrity and authenticity must be assured. |
|  | Examination | 1. M1: (Examination) – examine data collected to prove it is authentic or whether it has been tampered with. 2. M3: (Examination) –null; 3. M7: (Examination) –null; 4. M9: (Examination) – forensic examinations it's critical not to change any evidence. 5. M11: (Examination) - The digital examination begins with preserving the digital crime scene, dead or live analysis. | Examination is a process to ensure that the data collected is authentic and has not been tampered with. |
|  | Backup | 1. M1: (Backup) – The data that used to restore the database before intruder activity; 2. M7: (Backup) – Backup using to restore data dictionary; 3. M10: (Backup) – Collection of database objects that collected by MySQL utilities. 4. M14: (Backup) – backup the DBMS to protect the company's revenue from a DBMS failure. 5. M17: (Backup) – An exact copy of a former overall snapshot that can be played back efficiently as a whole. | Backup providing an exact copy of a former overall snapshot that can be played back efficiently as a whole. It can protect company's revenue from failure. |
|  | Transaction | 1. M1: (Transaction) – Intruder activities; 2. M10: (Transaction) – transaction that store in undo or redo log for recovery purpose. 3. M13: (Transaction) – Transactions are a group of database commands the user application perform it on the database, which insert, delete, and update the rows of the current state. 4. M19: (Transaction) - Transactions help manage concurrent access to the database and can be used for recovery. | Transaction is database commands the user application perform it on the database, which insert, delete, and update the rows of the current state. Transactions help manage concurrent access to the database. It store in undo or redo log for recovery purpose. |
|  | Transaction log | 1. M2: (Transaction logs) – The SQL Server transaction log contains a record of all insert, update and delete statements made within the database. 2. M12: (Transaction logs) – The transaction logs help to determine previously executed queries on the database and this is often helpful in various investigations. 3. M16: (Transaction logs) – source of evidence; 4. M18: (Transaction logs) - transaction log is useful for recovery from failed transactions and retrieval of a consistent version of the database in the event that the system crashes. | Transaction log is useful for recovery from failed transactions and retrieval of a consistent version of the database in the event that the system crashes. Transaction log help to determine previously executed queries on the database and this is often helpful in various investigations. |
|  | Investigation team | 1. M1: (Investigation team) –replay or reverse a series of changes involving any number of cells in a table, which may allow him or her to closely track the intruder’s actions. 2. M3: (Forensic examiner) – collect non-volatile evidence; 3. M4: (Investigation team) – null; 4. M5: (Investigation team) – investigator can get some information from reference table list with search with field attributes, check whether actual data of reference table match with investigation list or not, and join it to the worktable. 5. M6: (Examiner) - an examiner should receive a search warrant from the court. Then, he should secure the data resources of the company. 6. M7 : (Investigation team) – investigator searches data using powerful queries 7. M10: (Investigator) – identifying activity patterns and related database activity. 8. M12: (Investigation team) – An investigator has to be certain that an optimized query is an exact representation of the original query, especially when dealing with damaged databases where data files on the database might have been modified. 9. M13: (Investigation team) – investigator could compare the attempted SQL Server injection attacks identified within logs and memory dumps against actual statements executed within SQL Server to verify whether the attack code was correctly tunnelled and executed by the database. 10. M15: (Investigation team) – investigator can move on to collecting the evidence, preserving, and transporting the evidence. 11. M16: (Investigation team) - An investigator must determine which data are pertinent to an investigation in order to reduce the size of metadata. 12. M18: (Investigation team) – identify the changes made to compromise a database and when the changes were made during an analysis. | Investigation team are qualified and experience team who assigning by company or court to reveal database incident. An Investigation team must determine which data are pertinent to an investigation in order to reduce the size of metadata. Investigator could compare the attempted attacks identified within logs and memory dumps against actual statements executed within database. |
|  | Integrity | 1. M2: (Integrity) – copy of evidence should be a provably exact copy of the original data. 2. M12: (Integrity) - Ensure that the database has not been coerced into giving false result. 3. M14 :( Evidence Integrity) – Ensures that evidence results are not influenced by modifications made to the database. 4. M17: (Integrity) – Integrity of evidence. | Integrity ensures that evidence results are not influenced by modifications made to the database. The copy of evidence should be a provably exact copy of the original data. |
|  | Source | 1. M1: (Resources) – SQL cache, Undo log or Redo log. 2. M7: (Source) – the logs and the authorisation information. 3. M12: (Source) - sources of forensics data in databases consist of files storing histories relating to the database. Some of these files are specifically reserved for the database, for example the database log file and data files while others such as the web server logs and the system event logs of an operating system are not explicitly reserved for the database server usage. 4. M13: (Source) – Source of evidences and information. 5. M14: (Source) –Backup server for the DBMS where it can be proven that the backup server has not been compromised and we can trust the data model. 6. M16: (Source) – metadata sources in databases for capturing evidences by detecting major databases attacks and reconstructing the databases in case of anti-forensics attacks independent of the DBMS system used. | Sources of forensics data in databases consist of volatile and non-volatile files storing histories relating to the database. Some of these files are specifically reserved for the database. |
|  | Evidence | 1. M2: (Evidence) – evidences that used to prove compromised database; 2. M7: (Evidence) – data that exit on database on earlier time. 3. M9: (Evidence) – Evidence is most commonly found in files and databases that are stored on hard drives and storage devices and media. 4. M10: (Evidence) – collected data that submitted to court of law. 5. M11: (Evidence) – null; 6. M16: (Evidence) – Evidence is information stored or transmitted in binary form that may be relied on in court. The evidence will consist of who, why, when, what, how and where the malicious transaction were carried out. | Evidence is most commonly found in files and databases that are stored on hard drives and storage devices and media. Its information that stored or transmitted in binary form that may be relied on in court. It consists of who, why, when, what, how and where the malicious transaction were carried out. |
|  | Damaged database | 1. M1: (Damage Database ) – null; 2. M12: (Damaged database) - of damaged or destroyed databases refers to databases where the data contained or other data files may have been modified, deleted or copied from their original locations into other places. These databases may or may no longer be operational depending on the extent of the damage done. | Damaged or destroyed databases refers to databases where the data contained or other data files may have been modified, deleted or copied from their original locations into other places. These databases may or may no longer be operational depending on the extent of the damage done. |
|  | Modified Database | 1. M12: (Modified Database) – Modified database is a database which has not been compromised or damaged but has undergone changes due to normal business processes since the event of interest occurred. 2. M14: (Modified Database) - The modified database is a bit easier to determine because the investigator may have an idea of what has been done on the database after the event of forensic interest has occurred. The investigator can achieve this by considering the environment in which the database resides. Ultimately, the true dimensions of the database can only be determined after running some tests on the database. | Modified database is a database which has not been compromised or damaged but has undergone changes due to normal business processes since the event of interest occurred. Modified database is a bit easier to determine because the investigator may have an idea of what has been done on the database after the event of forensic interest has occurred. |
|  | Compromised database | 1. M12: (Compromised database) - Compromised database as a database where some of the metadata or some software of the database management system (DBMS) have been modified by an attacker even though the database is still operational. 2. M14: (Compromised database) - The second dimension of database forensics discussed here is compromised databases. Compromised databases represent DBMSs where the metadata or software of the DBMSs has been modified by an attacker. | Compromised database is a database where some of the metadata or some software of the database management system (DBMS) have been modified by an attacker even though the database is still operational. |
|  | Database administrator | 1. M11: (Database administrator) – They are responsible in overseeing and managing common resources used by multiple users within the organisation. The DBA is primarily responsible for authorising access to the database, coordinating and monitoring its use and for acquiring software and hardware resources. The DBA is also accountable for breach of security and database performances issues. 2. M16: (Database administrator) – is the one who monitors and maintains database in an organized way. But database forensics allows us monitoring the database along with tracing the actions of a DBA if he misses his access privileges. 3. M17: (Database administrator) - The database administrator is able to execute any kind of legal query, even those which may be considered malicious. 4. M19: (Database administrator) - null; | Database administrator is the one who monitors and maintains database in an organized way. But database forensics allows us monitoring the database along with tracing the actions of a DBA if he misses his access privileges. |
|  | Incident | 1. M2: (Event) – The event which added to timeline. 2. M3: (Incident) – An incident may be any action performed to compromise the confidentiality, availability and integrity of an information system. 3. M4: (Event) – null; 4. M9: (Event ) – The events which copied to the collection server for analysis 5. M10: (Event) – Notable events such as failed database login attempts, successful user logins, and anomalous database activity can be identified and added to an investigation timeline. 6. M7: (Incident) – Any action/event that corrupts the data accidentally or deliberately caused and compromises the confidentiality, availability and integrity of an information system. 7. M14: (Incident) – null; 8. M18: (Incident) – null; | Incident is an action/event that corrupts the data accidentally or deliberately caused and compromises the confidentiality, availability and integrity of an information system. Incident events copied to the forensic workstation for analysis. It includes changed database, compromised database, and destroyed database. |
|  | Database Server | 1. M1: (Database Server) – null; 2. M2: (Database Server) – null; 3. M3: (Database Server) – null; 4. M4: (Database Server) – Database server stores sensitive financial information, but it is configured with default database logging and no third-party logging solution is in place. 5. M5: (Database Server) – null; 6. M8: (Database Server) – Stored an important data. 7. M9: (Database Server) – null; 8. M10: (Database Server) – null; 9. M12: (Database Server) – null; 10. M15: (Database Server) – null; | Database server is a stand-alone computer in a local area network that holds and manages the database. It implies that database management functions such as locating the actual record being requested is performed in the server computer. |
|  | Output file | 1. M2: (Output file) – An output file was established to log the SQL statements and their associated results securely to my forensics workstation. 2. M3: (Output file) – All output from the live response tools should be written to a collection server across the network. 3. M8: (Output file) - When database files are acquired in a normal way, the contents of database should be output into CSV files that have readability and are easy to use | An output file is a collection of was established to log the SQL statements and their associated results securely to forensic workstation. Also the output from the live response tools should be written forensic workstation. |
|  | Log file | 1. M2: (Log file) – log files where the changes are written. 2. M9: (Log file) – null; 3. M10: (Log file) – Log files are most important data facts for investigation, as they contain the text of statements that include sensitive information such as passwords. 4. M12: (Database log file) – null; 5. M14: (Log file) – log files may reveal the compromise that has occurred in the original DBMS, and then the backup DBMS's log files can be checked to see if the same compromise has happened there. 6. M15 :( Log file) – one of the resources which used to track database attack; 7. M16: (Log file) – null; 8. M17: (Log file) – log files which contain a range of context and data of a database management system. Because of the architecture of MySQL it is possible to switch storage engines depending on the desired use case 9. M18: (Log file) – the log files keep record of the events that occur on the database | Log files are most important data facts for investigation, and one of the resources which used to track database attack. They contain the text of statements that include sensitive information such as passwords. Log files may reveal the compromise that has occurred in the original DBMS. |
|  | Database file | 1. M2: (Database files) – null; 2. M8: (Database files) – database file grasp from database server and imported in clean server to investigate it. 3. M16: (Database files) – Database files are logs contain useful information and it also contains routine operational data which may not be required at the time of analysis. 4. M19: (Database files) – null; | Database files are logs contain useful information and it also contains routine operational data which may not be required at the time of analysis. |
|  | Data file | 1. M2: (Data files) – where database store the events. 2. M3: (Data files) – the physical files for oracle. 3. M4: (Data files) – null; 4. M10: (Data files) – data files attached to a trusted forensic machine and used to support activity reconstruction artefact analysis. 5. M12: (Data files) – null; 6. M14: (Data files) – null; 7. M17: (Data files) – Databases store their content in so-called data files in the file system. Direct access to these files could be used in order to change data without invoking the DBMS, thus circumventing all logging- and monitoring mechanisms. 8. M18: (Data files) – The data files are used to store database objects such as tables and procedures. | The data files are used to store database objects and contents such as tables and procedures. Data files can also be attached to a trusted forensic machine and used to support activity reconstruction artefact analysis. |
|  | DBMS | 1. M4: (DBMS) – null; 2. M5: (DBMS) – Common DBMS offers DBMS system catalog information about database as to view format. 3. M7: (DBMS) - Define the DBMS from forensic perspective, where classified into four layers and three dimensions. 4. M12: (DBMS) – Discuss DBMS from three side’s dimensions, compromised, changed, and destroyed. 5. M15: (DBMS) –discuss DBMS from one perspective which is application layer 6. M17: (DBMS) - Database Management Systems (DBMS) provide mechanisms to ensure system integrity and to recover the database from inconsistent states or failures. 7. M19: (DBMS) – null; | Database management system is collection of programs that enable users to define, create, maintain and control access to a database by the use of various data management languages such as Data Definition Language (DDL), Data Manipulation Language (DML) and Structured Query Language (SQL). It consists of four abstract layers: a data model layer, a data dictionary layer, an application schema layer and an application data layer. |
|  | Incident responding | 1. M2: (Incident response )- null; 2. M4: (Incident responding) – Gathering incident details such as any information about incident events and known timelines, the parties involved thus far in the investigation, and the size and number of databases involved. The credential is required to login in high level for investigation. Also responding to an incident required avoiding any roadblock such windows firewall, network access control, IDS, IPS and versus | Incident responding means gathering incident details such as any information about incident events and known timelines, the parties involved thus far in the investigation, and the size and number of databases involved. The credential is required to login in high level for investigation. Also responding to an incident required avoiding any roadblock such windows firewall, network access control, IDS, IPS and versus |
|  | Company | 1. M3: (Organization) –null; 2. M4: (Organization) – null; 3. M5: (Company) – the owner of resources. 4. M6: (Company) – Victim. 5. M8: (Company)- Victim 6. M9: (Organization) – null; 7. M13: (Organization) – Owner of data. 8. M14: (Company) –null; 9. M15: (Company) –null; | The owner of resources. |
|  | Court | 1. M3: (Court) – null; 2. M6: (Court) – null; 3. M7: (Court) – null; 4. M15: (Court) – null; 5. M17: (Court) – null; 6. M10: (Court of law) – null; 7. M16: (Court of law) – null; | a formal legal meeting in which evidence about crimes, disagreements, etc., is presented to a judge and often a jury so that decisions can be made according to the law |
|  | Live response | 1. M3: (Live response) – Live Response is all about recovering and safely storing volatile data for later analysis, in other words, all the information that will disappear when the machine is disconnected from the network and switched off. Further, Live Response gives the forensic examiner the chance to collect non-volatile evidence in a human readable format. 2. M9: (Live response) – a Live Response is all about recovering and safely storing volatile data for later analysis, in other words, all the information that will disappear when the machine is dis-connected from the network and switched off. Further, Live Response gives the forensic examiner the chance to collect non-volatile evidence in a human readable. 3. M12: (Live response) – null; | Live Response is all about recovering and safely storing volatile data for later analysis, in other words, all the information that will disappear when the machine is disconnected from the network and switched off. Further, Live Response gives the forensic examiner the chance to collect non-volatile evidence in human readable format. |
|  | Forensic workstation | 1. M14: (Forensic environment) – classified the investigation environment to tow environment clean and found 2. M9: (Evidence collection server) – null; 3. M3: (Collection server) – the collection server can be a laptop but the incident responder should ensure that it has enough free space to hold all of the data which could run into several gigabytes. 4. M8: (Investigator computer) – the locations where the investigator did his investigation, he collected all database files, log file, imported files to this computer and install new system. 5. M2: (Trusted forensic workstation) – The outputs from the tools run during this investigation, will be saved on the trusted forensic workstation as opposed to the un-trusted target host. 6. M10: (Trusted forensic machine) – When table data is deleted, the data is hidden rather than actually being purged from the system. By analysing MySQL Server data files, you can often recover previously deleted table data. Data files can also be attached to a trusted forensic machine and used to support activity reconstruction artefact analysis. 7. M4: (Forensic Workstation) - it’s the environment that the investigator prepared it for forensic investigation locally or remotely. It includes trusted computer, install local SQL server instance and install SQL server workstation tools. | Forensic workstation is environment that the investigator prepared it for forensic investigation. It classified into tow environment clean and found environment. Furthermore, It’s a trusted environment, where collected all database files, log file, imported files copied. |
|  | Forensic Technique | 1. M5: (Investigation extraction methods) - Investigation extraction methods for database table relationship dividing application based method and database based method and analyse their strength and weakness. 2. M14: (Forensic Techniques) – forensic methods that could be used in various circumstances to conduct a data model sensitive investigation. Depending on the forensic environment, the DBMS investigator should understand the arguments for either a clean or found data model environment, and consequently select a method that is appropriate for the investigation. 3. M15: (Forensic Techniques) - forensic methods that could be used in various circumstances to conduct a data model sensitive investigation. Depending on the forensic environment, the DBMS investigator should understand the arguments for either a clean or found data model environment, and consequently select a method that is appropriate for the investigation. | Forensic Technique are a branch of database forensic investigation techniques that could be used in various circumstances such as segments a DBMS into four abstract layers, and to conduct sensitive investigation depending on the forensic environment which may be a clean or found environment. |
|  | Malicious Transaction | 1. M13: (Malicious transactions) – Malicious transactions may damage the database integrity and availability. 2. M16: (Malicious transactions) - Malicious command to cause harm. | Malicious transactions may damage the database integrity and availability. |
|  | Timeline | 1. M2: (Timeline) – Initial timeline will map out the notable digital events which have been identified thus far and establish an investigation scope which will be used during the Media Analysis phase. 2. M9: (Timeline) – null; 3. M10: (Timeline) – Notable events such as failed database login attempts, successful user logins, and anomalous database activity can be identified and added to an investigation timeline. This timeline will aid an investigator in identifying activity patterns and related database activity, which may not be sequentially logged within collected log files. 4. M17: (Timeline) – null; 5. M18: (Timeline) - creating timeline of events can assist an investigator to gain insight into the events that occurred and the people involved. It also assists in identifying patterns and anomalies that may reveal other sources of evidence in a system. | Timeline map out the notable digital events which have been identified thus far and establish an investigation scope which will be used during the analysis phase. Notable events such as failed database login attempts, successful user logins, and anomalous database activity can be identified and added to an investigation timeline. This timeline will aid an investigator in identifying activity patterns and related database activity, which may not be sequentially logged within collected log files. |
|  | Interview | 1. M6: (Interview) – To verify the existence of a server managed by the company, an interview with a high ranked company manager should be conducted. 2. M8: (Interview) - Interviews is very important step with the company staff member in charge of the system. In the stage of interview, it is likely to additionally grasp server locations and accounting information besides basic information such as IP of the database server and service port numbers. | Interview is important step with the company staff member in charge of the system and with a high ranked company manager. It’s very useful To verify the existence of a server managed by the company as well as grasp server locations and accounting information besides basic information such as IP of the database server and service port numbers. |
|  | Volatile artefact | 1. M4: (Volatile artefact) – Volatile artefacts are collections of related volatile database server and operating system artefacts such as memories artefacts which hold volatile data 2. M12: (Volatile artefact) – null; | Volatile artefacts are collections of related volatile database server and operating system artefacts such as memories artefacts which hold volatile data. |
|  | Non-volatile Artefact | 1. M4: (Non-volatile Artefacts) – Non-volatile artefacts are collections of related non-volatile database server and operating system artefacts such as database file, and OS log artefacts which hold non-volatile data. 2. M12: (Non-volatile Artefacts) – null; | Non-volatile artefacts are collections of related Non-volatile database server and operating system artefacts such as database file, and OS log artefacts which hold non-volatile data. |
|  | Decision | 1. M3: (Decision) – decision taken before an incident ever occurs whether to disconnect database server from the network or not. 2. M11: (Decision) – a decision is made to isolate the network, depending on the criticality of the incident. | Decision is made to isolate the network from database server, depending on the criticality of the incident. |
|  | Report | 1. M10: (Forensic report) – null; 2. M6: (Report) – is a document which secured and summited to court which hold data on accounting fraud cases. 3. M11: (Report) – null; 4. M16: (Final forensic report) – final report hold sequences of events and activities about users or system. | Report is a document which holds sequences of events, activities about users or system, and data on accounting fraud cases, which secured and summited to court. |
|  | Artefact | 1. M10: (Artefact) – Reside within operating system files and areas of memory that are explicitly reserved for SQL Server use. 2. M11: (Artefact) – An object that has been intentionally made or produced for a certain purpose. In reference to database, artefacts can be but not limited to event logs, data files, executable modules, registry values, settings, data cache holds recently access data from objects such as tables and indexes. SQL cache stores recently executed SQL statements and transaction log records change activity 3. M13: (Artefact) – provide valuable clues during a database investigation. These artefacts may hold clues that will help investigator piece together the incident events. So multiple evidences from various artefacts will capture. | Artefacts provide valuable clues during a database investigation. It may hold clues that will help investigator to reveal the incident events. It includes event logs, data files, executable modules, registry values, settings, data cache, and SQL cache. |
|  | Live Acquisition | 1. M4: (Live Acquisition) – is conducted using the resources and binaries of the target database server. These binaries can include system, network protocol, and SQL Server libraries, function, and procedures. 2. M12: (Live acquisition) – a live data acquisition occurs when the system being analysed is still running while the analysis is being performed. | A live data acquisition occurs when the system being analysed is still running while the analysis is being performed. It conducted using the resources and binaries of the target database server. These binaries can include system, network protocol, and SQL Server libraries, function, and procedures. |
|  | Dead Acquisition | 1. M4: (Dead acquisition) – performed on a dormant SQL Server that is not operational. M12: (Dead acquisition) - The dead acquisition method involves copying of data from the system being investigated without using the system itself. | The dead acquisition method involves copying of data from the system being investigated without using the system itself. |
|  | Hybrid Acquisition | 1. M4: (Hybrid acquisition) – combines key elements of both live and dead acquisition methods to give you the best of both worlds. It can be viewed as a typical dead acquisition that is performed after the live acquisition of volatile data. 2. M12: (Hybrid acquisition) - the hybrid data acquisition method combines the key elements of both live and dead acquisition methods. | Combines key elements of both live and dead acquisition methods to give you the best of both worlds. It can be viewed as a typical dead acquisition that is performed after the live acquisition of volatile data. |

- M equivalent Model
