# Supplementary material for: Development and validation of a Database Forensic Metamodel (DBFM)
Source: PLoS One. 2017 Feb 1;12(2):e0170793. doi: 10.1371/journal.pone.0170793 (PMC5287479; doi:10.1371/journal.pone.0170793)
Supplement: S5 Appendix III — (DOCX) [file pone.0170793.s005.docx]

**S5 AppendixIII.Table E. Validation Summary against Model Set V1.**

| **Set V1  Model** | **(A) SUPPORT Set V1 Concepts (DBFM Support Concept)** | | **(B) NOT SUPPORT Set V1 Concept (Phase)** | **(C) MODIFY change type: New DBFM Concept"** |
| --- | --- | --- | --- | --- |
| Set V1(1):  [[1](#_ENREF_1)] | - Audit Trail Database   (*NonvolatileArtefact)*   - Activities (*IntruderActivity,TransactionLog*) - Database Triggers (*ForensicTechnique*) - Audit Information (*DataCollected*) - Administrator (*DatabaseAdministrator*) - Protect AuditLog Table   (*Integrity*)   - Insert Operations ( *IntruderActivity, TransactionLog*) - Detect Tampering (*Examination, IncidentResponding*) | - Tool (*ForensicTechnique)* - Modifications (*IntruderActivity*) - Original Data   (*DataCollected)*   - SQL Statements   (*TransactionLog*)   - AuditLog (*ForensicTechnique*) - Hashing (*Hashing*) - Audit Log Table   (*Artefact*) | All-supported | No |
| Set V1(2):  [[2](#_ENREF_2)] | - SQL Query (*ForensicTechnique*) - Server (*DatabaseServer*) - Cost-Based Optimizer (*ForensicTechnique*), - Information (*Evidence*), - Forensic examiner (*InvestigationTeam*) - Incident responder (*DatabaseAdministrator*) - Database Server (*DatabaseServer*), - Timeline (*Timeline*) - Event (*Incident*) - Dumping (*Backup*) - Dump data (*DataCollected*), - Backups (*Backup*) - Intrusion (*Incident*) - Examination (*Examination*), - PL/SQL injection (*Incident*) - Table (*Non-volatileArtefact*) | - Delete (*IntruderActivity,TransactionLog*) - Evidence (*Evidence*) - Redo logs (*RedoLog*) - Data files (*Databasefile*) - Views (*Artefact*) - Attacker’s activities (*IntruderAativity*) - Cache (*VolatileArtefact*) - Automatic Workload Repository (*ForensicTechnique*) - Actions (*Incident*), - Attacker’s actions (*IntruderAativity*) - Capture (*Capture*) - Organization’s database (*DatabaseServer*) - DBA (*DatabaseAdministrator*) | All-supported | no |
| Set V1(3):  [[3](#_ENREF_3)] | - Evidence (*Evidence*) - *Illegal Activities* (*IntruderActivity)* - Company (*Company*) - Investigators (*InvestigationTeam*) - Financial fraud (*IntruderActivity*) - Financial Statements (*TransactionLog*) - Obtain the database *(Capture)* - Database Administrator (*DatabaseAdministrator*) | - Database system   (*DatabaseManagementSystem*)   - Secret information (*TransactionLog*) - Crime (*Incident*) - Report (*Report*) - Law court *(court)* - Database Management   System (*DatabaseManagementSystem*)   - Digital forensic techniques (*ForensicTechnique*) | All-supported | No |
| Set V1(4):  [[4](#_ENREF_4)] | - Reconstruction (*Reconstruction*) - Query log   (*TransactionLog*)   - Desired value (*DataCollected*) - Actual query   (*ForensicTechnique*)   - Timeline (*Timeline*) | - Results (*Evidence*) - Relation (*Incident*) - Reconstructing values - (*Timeline*) - Operations (*TransactionLog*) - Desired Information (*DataCollected*) - Particular value (*Evidence*) | Reconstruction Algorithm, Searching | -Add:  “ReconstructionAlgorithm”  - Add: “Seraching” |
| Set V1(5):  [[5](#_ENREF_5)] | - Digital Signature   (*Hashing*)   - Verification (*IncidentVerfication*) - Master data (*DataCollecetd)* - Auditors (*InvestigationTeam*) - Data Owner (*Company*) - Specific Location (*DatabaseServer*) - Transaction (*TransactionLog*) - Database   (*DatabaseServer*) | - Validator (*ForensicTechnique*) - Hashing (*Hashing*) - Transaction data (*TransactionLog*) - Organization (*Company*) - Avalanche *(Incident)* - Security master database   (*DatabaseServer*)   - Tamper Detection (*Examination, IncidentResponding*) - Tampered Data (*IntuderActivity*) | All-supported | No |
| Set V1(6):  [[6](#_ENREF_6)] | - Data model (*DBMS*) - DBMS (*DatabaseManagementSyetm*) - Copy Evidence (Backup) - Original Copy (*DataCollecetd)* - Server (*DatabaseServer*) - Malicious tasks   (*IntruderActivity*)   - Hide data (*IntruderActivity)* - Backup Server (*Backup*) - Investigator (*InvestigationTeam*) - Resource *(Source)* - On-site Investigation (*FoundEnvironment*) | - Acquiring access (*Capture*) - Password guessing (*Incident*) - TNS Listener Exploits *(Incident)* - Database Administrator Logs *(Transactionlog*) - Forensic evidence (*Evidence)* - Evidence Collection (*DataAcquisition*) - Encase toolkit *(ForensicTechnique)* - Extract Evidence (*DataAcquisition*) - Mirrored (*Backup*) | Clean environment,  Found environment, Copying File | - Add: “CleanEnvironment”,  - Add: “Found Environment”,  - Add: “CopyingFile” |
| Set V1(7):  [[7](#_ENREF_7)] | - InnoDB *(NonovolatileArtefact*), - Log files(*LogFile*) - Data Files (*DatabaseFile*) - Log data (*TransactionLog*) - Previous Changes (*DatabaseActivity*) - Database Server (*DatabaseServer*) - Reconstruct previous queries (*Reconstruction*) - Checksum (Hashing), - Verification (*IncidentResponding*) - Delete(*DatabaseActivity*) | - Reconstructing Insert Statements (*Reconstruction*) - Table (*Non-volatile artifatc*) - Update (*DatabaseActivity*) - Log entries (TransactionLog) - Harvest (*Capture*) - Data manipulation statement   (*TransactionLog)*   - Query Reconstruction (*Reconstruction*) - Insert (*TransactionLog*) | All-supported | no |
| Set V1 (8):  [[8](#_ENREF_8)] | - Database Server (*DatabaseServer*) - Tampering (*Incident*) - Validator (*ForensicTechnique*) - Database Administrator (*DatabaseAdministrator*) | - Investigator Engine (*ForensicWorkstation)* - MySQL xml log (*TransactionLog*) - Hashing algorithm (*ForensicTechnique)* - Source (Source) - Tamper detection (*Examination, IncidentResponding*) | All-supported | No |

- Add equivalent addition

References

1. Basu, A., *Forensic tamper detection in SQL server*, 2006.

2. Litchfield, D., *Oracle forensics part 5: Finding evidence of data theft in the absence of auditing.* NGSSoftware Insight Security Research (NISR), Next Generation Security Software Ltd., Sutton, 2007.

3. Lee, G.T., et al. *Discovering Methodology and Scenario to Detect Covert Database System*. in *Future Generation Communication and Networking (FGCN 2007)*. 2007. IEEE.

4. Fasan, O.M. and M. Olivier, *Reconstruction in database forensics*, in *Advances in Digital Forensics VIII*. 2012, Springer. p. 273-287.

5. Abhonkar, P.D. and A. Kanthe, *Enriching Forensic Analysis process for Tampered Data in Database.*

6. Beyers, H., M.S. Olivier, and G.P. Hancke. *Arguments and Methods for Database Data Model Forensics*. in *WDFIA*. 2012.

7. Frühwirt, P., et al., *InnoDB database forensics: Enhanced reconstruction of data manipulation queries from redo logs.* Information Security Technical Report, 2013. **17**(4): p. 227-238.

8. Kambire, M.K., et al., *An Improved Framework for Tamper Detection in Databases.*
