# Supplementary material for: Development and validation of a Database Forensic Metamodel (DBFM)
Source: PLoS One. 2017 Feb 1;12(2):e0170793. doi: 10.1371/journal.pone.0170793 (PMC5287479; doi:10.1371/journal.pone.0170793)
Supplement: S6 Appendix III — (DOCX) [file pone.0170793.s006.docx]

**S6 AppendixIII.Table F Validation Summary against Model Set V2.**

| **Set V2  Model** | **Set V2 Concepts** |
| --- | --- |
| Set V2(1):  [[1](#_ENREF_1)] | Company, LogMiner, Redo logs, Reconstruct changes, SQL statements, Main machine, Source, Forensic analyst, Documentation, Incident, Timeline, Backup, Location, Command, Duplicate copy, Checksum, Integrity, Corruption, Recover the database, DBA, Database systems, Query the database, Events, Investigator, Separate server, Oracle versions Match, Test lab, Network, Temperature, Humidity, Atmospheric pressure, Heavy magnetism, Report, Hash, Hash values, Windows express tool, Solaris machine, Forensics analysis tool, Archiving the redo logs, Copy of redo log, Evidence, Law enforcement, Court, Airtight bag, Transactions, Analysis, Searching, Data files, Trigger, Destroyed database, Log file, Represent the information, Decision |
| Set V2(2):  [[2](#_ENREF_2)] | Data tampering, Unauthorized access, Malicious data modifications, Forensic analysis, Data breach, Bank information system, Clients banking accounts, Persecution department, Team of digital forensics, Collect all evidence, Suspicious transactions, Examine Suspicious transactions, Report, Court of low, Valid court evidence, Collecting data, Copying, Corruption event, Intrusion, Human intervention, DBMS, Hardware failure, Corruption time, Digital evidence, Audit logging, Intruder, Reconstruct, Data collected, Collects basic data, Integrity, Activities, Identity, Hash checksum, Identity of user, Tampered data, DBA, Transaction, Detecting data modification, SQL triggers, Event, Database tables, Hashed values |
| Set V2(3):  [[3](#_ENREF_3)] | Audit logging, Collected data, Collecting data, User activity, Digital evidence, Basic data, Actions, Information systems, Forensic analysis, Triggers, Log file, Backup, Replication, Data modification, SQL statements, Transaction, Detecting data modification, Event, Relational Database Management System, Database tables, Administrator, Evidence collected, Data validity, Data tampering, Delete record, Operation, Data integrity, Database engine, Transaction log, Database system, Disaster policy, Log backups, Illegal activity, Modification, Malicious person, Copying file |
| Set V2(4):  **[**[**4**](#_ENREF_4)**]** | Selection database, Network, Create database, Backup of database, Hackers, Database attack, Restore database, Transaction, Integrity, Application layer, Source, Organization, Destroy database, Database Management System, Command, Malicious code, Database server, Database forensic tools, Collecting sensitive information, Data definition language operations, Data manipulation language operations, Forensic analysis, Forensic techniques, Data analysis, Investigator, Database files, Server RAM, Evidences of database, Hash , Court of law, Decision, Test environment, Forensic examination, Data acquisition, IT administrators, Extraction of columns, Detection of Security Breach, Retracing user DML and DDL operations, Intruder Detection Systems |
| Set V2(5):  [[5](#_ENREF_5)] | Forensic comparison tool, Forensic examiner, Metadata, Assembling metadata, Forensic examination, Data preservation, Data analysis, Preserved data, DBMS, Suspect DBMS, Clean DBMS, Forensic tool, Assembled data, Integrity, Hash, Report, Commands, Mirrored suspect DBMS, Evidence, Testing environment. |
| Set V2(6):  [[6](#_ENREF_6)] | Financial frauds, Forensic auditors, Fraud discovering, Databases Files, Sources, Required files, Collecting, Events, Transactions, Metadata, Accounting databases, Data Preparation, Analysis, Techniques, Validation, Production data, Data Cleaning, Transformation, Suspicious transactions, Selection of Techniques, Searching, Data analysis, Auditors’ traditional investigative techniques, Gather evidences, Evidence, Interview, Documents, Anomalies, Organization, Capture |
| Set V2(7):  [[7](#_ENREF_7)] | Incident, Money laundering, Financial institution, Policies, Transactions, Suspicious, Evidence, Sources, Case Analysis, Preserving events, Examination, Data Acquisition, Organisation, De-normalised table, Information extracted, MySQL Server, Triggers, Extraction, Log files, Database queries, Database logs, Suspicious database logs, Frauds, Database activities, Data Examination, Selectively imaging, Database server, FTK imager, Review, Extracted database logs, Data Analysis, Digital forensic analysis tool, Data Presentation, Report, Recovered evidence |
| Set V2(8):  [[8](#_ENREF_8)] | Database Server, Authorized user, Modification, Forensic Analysis Algorithm, Tiled Bitmap forensic analysis algorithm, Forensic Analysis method, Forensic Analysis, Collects data, Evidence, Data dictionary, Attacker, Audit log, Metadata, Data mining tool, Database operation, DBMS, Transaction, Hash, Intruder, Hashed value, Rehash Process, VALIDATOR, Old hash values, Tamper Detection, Tampering, Validation, Trigger, Event, Database Administrator, Master data, Location |
| Set V2(9):  [[9](#_ENREF_9)] | Investigator, Database management system, Stored data, History file, Command, Program files, Copying, New instance of MySQL, Original instance of MySQL, Data files, Unauthorized persons, System administrator, System files, InnoDB, Individual database, Location, Reconstruct Databases, Metadata, Table data, Crash, Source files, Clean instance, Source machine |

1. Wright, P.M. *Oracle database forensics using LogMiner*. in *June 2004 Conference, SANS Institute*. 2005.

2. Azemović, J. and D. Mušić. *Efficient model for detection data and data scheme tempering with purpose of valid forensic analysis*. in *2009 International Conference on Computer Engineering and Applications (ICCEA 2009)*. 2009.

3. Azemovic, J. and D. Music. *Methods for Efficient Digital Evidences Collecting of Business Proceses and Users Activity in eLearning Enviroments*. in *e-Education, e-Business, e-Management, and e-Learning, 2010. IC4E'10. International Conference on*. 2010. IEEE.

4. Fatima, F., *Detecting Database Attacks Using Computer Forensics Tools*, 2011, Texas A&M University-Corpus Christi.

5. Beyers, H., M.S. Olivier, and G.P. Hancke. *An approach to examine the Metadata and Data of a database Management System by making use of a forensic comparison tool*. in *ISSA*. 2011. Citeseer.

6. Panigrahi, P.K. *A framework for discovering internal financial fraud using analytics*. in *Communication Systems and Network Technologies (CSNT), 2011 International Conference on*. 2011. IEEE.

7. Flores, D., O. Angelopoulou, and R.J. Self. *Combining Digital Forensic Practices and Database Analysis as an Anti-Money Laundering Strategy for Financial Institutions*. in *Emerging Intelligent Data and Web Technologies (EIDWT), 2012 Third International Conference on*. 2012. IEEE.

8. Gawali, P.P. and D.S.R. Gupta, *Database Tampering and Detection of Data Fraud by Using the Forensic Scrutiny Technique.* International Journal of Emerging Technology and Advanced Engineering.

9. Lawrence, A.C., *Forensic Investigation of MySQL Database Management System*, 2014.
