# Supplementary material for: Development and validation of a Database Forensic Metamodel (DBFM)
Source: PLoS One. 2017 Feb 1;12(2):e0170793. doi: 10.1371/journal.pone.0170793 (PMC5287479; doi:10.1371/journal.pone.0170793)
Supplement: S7 Appendix III — (DOCX) [file pone.0170793.s007.docx]

**S7 AppendixIII.Table G. Frequency Results of Identification and Artefact Collection-process concepts.**

| **No** | **DBFM 1.1 Concepts** | **Model Set V2** | | | | | | | | | **Frequency of Concept** |
| --- | --- | --- | --- | --- | --- | --- | --- | --- | --- | --- | --- |
|  |  | **1** | **2** | **3** | **4** | **5** | **6** | **7** | **8** | **9** |  |
| **IDENTIFICATION CONCEPTS** | | | | | | | | | | | |
|  | Company | √ |  |  | √ |  | √ | √ |  |  | 4 |
|  | ForensicWorkstation | √ |  |  | √ | √ |  | √ |  | √ | 5 |
|  | CleanEnvironment | √ |  |  | √ | √ |  |  |  | √ | 4 |
|  | FoundEnvironment |  |  |  |  | √ |  | √ |  | √ | 3 |
|  | InvestigationTeam | √ | √ |  | √ | √ | √ |  |  | √ | 6 |
|  | ForensicTechnique | √ | √ | √ | √ | √ | √ | √ | √ |  | 8 |
|  | Source | √ | √ | √ | √ | √ | √ | √ | √ | √ | 9 |
|  | Artefact | √ | √ | √ |  | √ | √ | √ |  | √ | 7 |
|  | VolatileArtefact | √ |  |  | √ |  |  |  |  |  | 2 |
|  | NonvolatileArtefact | √ | √ | √ |  | √ | √ | √ |  | √ | 7 |
|  | DatabaseFile | √ |  |  | √ |  | √ |  |  | √ | 4 |
|  | LogFile | √ |  | √ | √ |  | √ |  |  | √ | 5 |
|  | UndoLog |  |  |  |  |  |  |  |  |  | 0 |
|  | DamagedDatabase | √ |  |  | √ |  |  |  |  |  | 2 |
|  | ModifiedDatabase |  |  | √ |  |  |  |  | √ |  | 2 |
|  | CompromisedDatabase |  | √ |  | √ | √ |  |  |  |  | 3 |
|  | DatabaseAdministrator | √ | √ | √ | √ |  |  |  | √ | √ | 6 |
|  | Incident | √ | √ | √ |  | √ | √ | √ | √ | √ | 8 |
|  | DatabaseServer | √ |  | √ | √ |  |  | √ | √ |  | 5 |
|  | DatabaseManagementSystem | √ | √ | √ | √ | √ |  |  | √ | √ | 7 |
|  | IncidentResponding | √ | √ | √ | √ |  | √ |  | √ | √ | 7 |
|  | Interview |  |  |  |  |  |  | √ |  |  | 1 |
|  | LiveResponse | √ |  |  |  |  |  |  |  |  | 1 |
|  | Capture | √ |  |  |  |  | √ |  |  |  | 2 |
|  | Decision | √ |  |  | √ |  |  |  |  |  | 2 |
|  | Report | √ | √ |  |  | √ |  | √ |  |  | 4 |
| **ARTEFACT COLLECION CONCEPTS** | | | | | | | | | | | |
| 1. 1 | ForensicWorkstation | √ |  |  | √ | √ |  | √ |  | √ | 5 |
|  | Source | √ | √ | √ | √ | √ | √ | √ | √ | √ | 9 |
|  | ForensicTechnique | √ | √ | √ | √ | √ | √ | √ | √ |  | 8 |
|  | FoundEnvironment |  |  |  |  | √ |  | √ |  | √ | 3 |
|  | CleanEnvironment | √ |  |  | √ | √ |  |  |  | √ | 4 |
|  | Artefact | √ | √ | √ |  | √ | √ | √ |  | √ | 7 |
|  | NonVolatileArtefact | √ | √ | √ |  | √ | √ | √ |  | √ | 7 |
|  | VolatileArtefact | √ |  |  | √ |  |  |  |  |  | 2 |
|  | DatabaseFile | √ |  |  | √ |  | √ |  |  | √ | 4 |
|  | LogFile | √ |  | √ | √ |  | √ |  |  | √ | 5 |
|  | UndoLog |  |  |  |  |  |  |  |  |  | 0 |
|  | InvestigationTeam | √ | √ |  | √ | √ | √ |  |  | √ | 6 |
|  | Report | √ | √ |  |  | √ |  | √ |  |  | 4 |
|  | DataAcquisition | √ |  |  | √ |  |  | √ |  |  | 3 |
|  | DataCollected | √ | √ | √ |  | √ |  | √ | √ |  | 6 |
|  | LiveAcquisition | √ |  |  |  |  |  |  |  |  | 1 |
|  | DeadAcquisition | √ |  |  |  |  |  |  |  |  | 1 |
|  | HybridAcquisition |  |  |  |  |  |  |  |  |  | 0 |
|  | Hashing | √ |  |  | √ | √ |  |  | √ |  | 4 |
|  | Integrity | √ | √ | √ | √ | √ | √ | √ |  |  | 7 |
|  | OutputFile |  |  |  |  |  |  |  |  |  | 0 |
|  | Backup | √ |  | √ | √ |  |  |  |  |  | 3 |
