# Supplementary material for: Development and validation of a Database Forensic Metamodel (DBFM)
Source: PLoS One. 2017 Feb 1;12(2):e0170793. doi: 10.1371/journal.pone.0170793 (PMC5287479; doi:10.1371/journal.pone.0170793)
Supplement: S8 Appendix III — (DOCX) [file pone.0170793.s008.docx]

**S8 AppendixIII.Table H. Frequency Result of Artefact Analysis and Documentation & Presentation-process concepts.**

| **DBFM 1.1 Concepts** | | **Model Set V2** | | | | | | | | | **Frequency of Concept** |
| --- | --- | --- | --- | --- | --- | --- | --- | --- | --- | --- | --- |
|  |  | **1** | **2** | **3** | **4** | **5** | **6** | **7** | **8** | **9** |  |
| **ARTEFACT ANALYSIS CONCEPTS** | | | | | | | | | | | |
|  | ForensicWorkstation | √ |  |  | √ | √ |  | √ |  | √ | 5 |
|  | CleanEnvironment | √ |  |  | √ | √ |  |  |  | √ | 4 |
|  | FoundEnvironment |  |  |  |  | √ |  | √ |  | √ | 3 |
|  | DatabaseManagementSystem | √ | √ | √ | √ | √ |  |  | √ | √ | 7 |
|  | Reconstruction | √ | √ |  | √ |  |  |  |  | √ | 4 |
|  | TimeLine | √ |  |  |  |  |  |  |  |  | 1 |
|  | InvestigationTeam | √ | √ |  | √ | √ | √ |  |  | √ | 6 |
|  | Report | √ | √ |  |  | √ |  | √ |  |  | 4 |
|  | Evidence | √ | √ | √ | √ | √ | √ | √ | √ |  | 8 |
|  | IntruderActivity | √ | √ | √ | √ |  | √ | √ | √ |  | 7 |
|  | ForensicTechnique | √ | √ | √ | √ | √ | √ | √ | √ |  | 8 |
|  | Searching | √ |  |  |  |  | √ |  |  |  | 2 |
|  | CopyingFile | √ | √ | √ |  | √ |  |  |  | √ | 5 |
|  | ReconstructionAlgorithm |  |  |  |  |  |  |  | √ |  | 1 |
|  | Examination |  | √ |  | √ | √ |  | √ | √ |  | 5 |
|  | DataCollected | √ | √ | √ |  | √ |  | √ | √ |  | 6 |
|  | DatabaseFile | √ |  |  | √ |  | √ |  |  | √ | 4 |
|  | TransactionLog | √ |  | √ | √ |  | √ |  | √ |  | 5 |
| **DOCUMENTATION & PPRESENTATION CONCEPTS** | | | | | | | | | | | |
| 1 | Court | √ | √ |  | √ |  |  |  |  |  | 3 |
| 2 | Company | √ |  |  | √ |  | √ | √ |  |  | 4 |
| 3 | InvestigationTeam | √ | √ |  | √ | √ | √ |  |  | √ | 6 |
| 4 | Report | √ | √ |  |  | √ |  | √ |  |  | 4 |
| 5 | Evidence | √ | √ | √ | √ | √ |  | √ | √ |  | 7 |
